# Tracing multi-isotopically labelled CdSe/ZnS quantum dots in biological media

**DOI:** 10.1038/s41598-020-59206-w

**Published:** 2020-02-18

**Authors:** N. Izyan Supiandi, G. Charron, M. Tharaud, M. F. Benedetti, Y. Sivry

**Affiliations:** 1 Institut de Physique du Globe de Paris, Sorbonne Paris Cité, Univ. Paris Diderot, UMR 7154, CNRS, F-7, 5005 Paris, France; 2 Laboratoire Matière et Systèmes Complexes (MSC), Université Paris Diderot, 75013 Paris, France

**Keywords:** Chemical tools, Mass spectrometry

## Abstract

The strengths and limits of isotopically labelled Engineered Nanoparticles (spiked ENPs) spread in biological media have been assessed. Multi-spiked CdSe/ZnS quantum dots (QDs), measuring 7 nm and coated with thioglycolic acid (TGA), were synthesized and enriched in ^68^Zn, ^77^Se and ^111^Cd. These QDs were dispersed at very low concentrations (0.1 to 5000 ppt) in diverse biological matrices (synthetic saliva, synthetic urine, plasma and Dulbecco’s phosphate buffered saline - DPBS growth medium) and the isotopic compositions were determined by HR-ICP-MS. The initial QDs concentrations were calculated to assess the limit of quantification (QD-LOQ) according to the matrix and the isotopically enriched element. The obtained results demonstrated the advantages of the isotopic labelling method in order to work at very low concentrations: the QD-LOQ values for the spiked Zn, Cd and Se originated from the QDs were 10, 0.3 and 6 ppt, respectively, which is below the conventional LOQ of the HR-ICP-MS used (30, 3 and 60 ppt for Zn, Cd and Se, respectively). Conversely, in complex matrices such as saliva, urine, plasma and DPBS growth medium, the QD-LOQ values increased significantly, with values ranging from 16 to 32 ppt for Cd, 446 to 10598 ppt for Zn and 1618 to 8317 ppt for Se. These QD-LOQs are dependent on factors as the elemental background concentration already present in the matrices, and the dilution factor. In this study, the QD-LOQs are expressed for the first time with respect to the background concentration in biological media (QD-RLOQ), which can be used to better assess and then predict the efficiency of the spiking method.

## Introduction

Quantum dots (QDs) are semiconductor nanocrystals with a diameter of 2–10 nm that possess unique optical and electrical properties *e.g*. photoluminescence and conductivity^1,2^. For example, the photoluminescence of QDs emitting in the visible region can be modified by changing their size and composition^3^. At a specific wavelength, bigger QDs emit longer wavelengths thereby resulting in red or orange emission colors, while smaller QDs emit shorter wavelengths (blue or green). They have been the focus of substantial attention over the past decade and are widely used in various applications: nowadays, QDs are mostly used in Light-Emitting Diodes (LEDs)/Organic LEDs (OLED) as light conversion materials (90%) and in laboratories for imaging (10%)^4,5^. Since the early 2000s, CdSe/ZnS QDs have been used in laboratories for disease sensing and the delivery of antibacterial/anticancer drugs^6–8^. However, the core of the QDs is traditionally composed of semiconductor solids such as CdS, CdSe and CdTe^9^ whereas Cd, which is known to be toxic, may be released after dissolution of the core. It is still a challenge to understand the physical and chemical behavior of QDs so as to be able to study their toxicity in complex biological matrices. The fluorescence of QDs, which is easily observed at high concentrations (ppm), cannot be observed at lower concentrations (ppt-ppb level), which is relevant for concentrations obtained after the dilution of the ENPs in biological fluids or after the partial dissolution of the ENPs^10^. At this point, the behavior of the QDs should be traced using alternative method such as atomic spectrometry which can be used to trace QDs at low concentrations in biological matrices without depending on their fluorescence. However, biological matrices are known to naturally contain a certain amount of trace metals such as Zn, Se, Fe and Mg, *e.g*. human urine contains *ca*. 650, 0.2 and 40 ppb of Zn, Cd and Se and human blood plasma contains *ca*. 1000, 0.3 and 100 ppb, respectively^11–13^.

Therefore, during fate and/or toxicity studies, it is crucial to distinguish the trace metals naturally present in the matrix from the ones originated from the QDs. Hence, we propose the use of isotopically labelled^14^ or “spiked” QDs coupled with High Resolution Inductively Coupled Plasma Mass Spectrometry (HR-ICP-MS) to trace the QDs and to understand their behavior in biological environments at low concentrations (ppt-ppb level). In fact, isotopically labelled nanoparticles (NPs) are increasingly being used in the literature to better quantify manufactured NPs in biological matrices^15–19^. For instance, Khan *et al*. showed the ability to conduct environmentally relevant exposures (20 µg ^68^Zn/L) of spiked ZnO NPs in order to study their bioavailability to estuarine snails, whereas Bourgeault *et al*. provided evidence for the critical use of spiked ^47^TiO_2_ NPs when studying the bioaccumulation of NPs at environmental concentrations (7–120 µg/L of ^47^TiO_2_ NPs) in zebra mussels in water. However, the concentrations of spiked NPs used in some other studies were still within the ppb to ppm range, which is higher than the relevant concentration to which living organisms could be exposed^20^. Furthermore, the limitations of the isotopic labelling technique for NPs in biological matrices have never been studied. In other words, despite the attractiveness of this cutting-edge method, the following still need to be determined: the extent to which this method can be used to study the fate and toxicity of QDs in a biological matrix and the lowest concentration that can be reliably used.

Therefore, the objective of this paper is to determine the limitations of the isotopic labelling technique for ENPs in biological matrices, which should be an important step prior to any future fate and toxicity study. Multi-spiked quantum dots with a CdSe/ZnS core/shell structure were synthesized and dispersed at very low and relevant concentrations (ppt-ppb) in various biological matrices (blood plasma, urine, saliva and growth medium). Measurements of the isotopic compositions by HR-ICP-MS and statistical data treatment were used to accurately determine the limits of quantification of the method (QD-LOQ), based on the isotopically labelled element and the matrix composition. This work is expected to provide a valuable basis for all future studies aimed at using isotopically labelled ENPs to determine their fate or toxicity in biological media.

## Experimental Section

### Synthesis of multi-isotopically labelled (i.e. multi-spiked) CdSe/ZnS quantum dots

#### Chemicals

Chemicals with a natural isotopic composition were purchased from Sigma Aldrich: chloroform (CHCl_3_, 99%), 1-octadecene (ODE, 90%), oleic acid (OA, technical, 90%), sulfur (S, 99.9%, powder), trioctylphosphine (TOP, 90%) and thioglycolic acid (TGA, 99%). Chemicals with a modified isotopic composition were purchased from ISOFLEX USA: zinc oxide (ZnO powder) enriched to 99.16% in ^68^Zn, cadmium oxide (CdO powder) enriched to 96.00% in ^111^Cd and selenium (Se powder) enriched to 99.20% in ^77^Se.

#### Synthesis

The QDs were manufactured with an adaptation of the protocol given by Bae *et al*.^21^. The modification consisted of substituting ^68^Zn-enriched ZnO for the initial zinc acetate precursor which was not available with a suitable modified isotopic composition. The details of the modified synthesis protocol as well as its functionalization by thioglycolic acid, TGA, to produce water-soluble QDs (TGA-coated QDs) are described in the Supporting Information (SI). The resulting characterization of the multi-spiked QDs will be compared to the QDs synthesized following the original protocol given by Bae *et al*. without modification of the isotopic composition, *i.e*. non-spiked QDs.

### Quantum dot characterization

#### Optical characterization

The UV-Vis absorption spectra of the isotopically modified (multi-spiked) TGA-coated QD stock solution with a UV-Vis spectrometer (Thermo Scientific Evolution 600). The emission spectrum was recorded using a spectrofluorometer (Horiba Scientific FluoroMax-4) at an excitation wavelength of 400 nm, and the fluorescence color emitted by the QD stock solution was also observed using a Vilber Lourmat compact transilluminator (BTCP-20.MC) at 312 nm. The characterization results for the non-spiked QDs synthesized using the original protocol are used as a comparison.

#### Physical properties

The size and shape of the multi-spiked QDs were observed by Transmission Electron Microscopy (TEM) using a JEOL 2100 F electron microscope operating at 200 kV and equipped with a field emission gun, a high-resolution UHR pole piece and a Gatan GIF 200 l imaging filter: the diluted suspensions of the QDs were deposited on a copper grid for this observation. The TEM pictures were processed with the software ImageJ 1.51n.

#### Chemical analysis

The microscope used above was coupled with electron-dispersive X-ray spectroscopy (EDXS) using a JEOL detector with an ultrathin window allowing the detection of low atomic mass elements to perform a chemical analysis on the QDs. To measure the total concentrations of Cd, Se and Zn in the TGA-coated QD stock solution, diluted QD solutions (dilution factor: 500 and 1000) were analyzed by ICP-OES using the Thermo Scientific iCAP 6000 Series after complete acid digestion with HF/HNO_3_ (SI) and then after simple acidification (2% nitric acid) to evaluate the best sample preparation (*i.e*. complete digestion) before analysis. Their isotopic composition was verified by HR-ICP-MS using Thermo Scientific Element II.

### Choice of biological matrices

The choice of blood plasma, urine, saliva and growth medium was based on the numerous studies that use QDs, *e.g*. in bio-imaging and bio-sensing, as described previously. The composition of the chosen biological matrices is described below.

#### Artificial urine

The artificial urine recipe was prepared according to Shiotsuki *et al*.^22^ (Table S1, SI). Zn, Cd and Se with natural isotopic compositions were also added into the solution to obtain the average concentrations found in human urine of 650, 0.2 and 40 ppb, respectively^11–13^.

#### Artificial saliva

The artificial saliva recipe is adapted from various studies^23–25^ (Table S2, SI). Cd and Se with natural isotopic compositions were also added into the solution given that their usual concentrations in human urine are 0.5 and 3 ppb, respectively^26,27^. Zn was not added to the solution as it already contains 220 ppb of this element coming from porcine mucin; this value is consistent with the typical Zn concentration in human saliva (260 ppb)^26^.

#### DBPS growth medium

Dulbecco’s phosphate buffered saline (DPBS) was prepared by following the recipe from Dulbecco and Vogt^28^ (Table S3, SI). The concentrations of Zn and Se in the medium were 7700 and 1580 ppt respectively, while the Cd concentration was lower than the ICP-MS LOQ.

#### Rat plasma

Rat plasma (adults, male, Sprague Dawley) was purchased from Janvier Labs, France. The concentrations of Zn, Cd and Se were 1020 000, 45 and 620 000 ppt, respectively. The plasma was stored at −18 °C and thawed at room temperature before use.

### Model for QD dissemination in biological matrices

The laboratory glassware and materials used in these experiments *e.g*. PP tubes, bottles and pipette tips were washed with HCl 1 N to eliminate contamination by trace metals, especially Zn, Cd and Se.

TGA-coated CdSe/ZnS quantum dots were added separately in all four selected biological matrices (rat plasma, DPBS solution, artificial urine and saliva solutions at target concentrations of 0.1, 10, 50, 100, 1000 and 5000 ppt of Zn originated from the QDs, resulting in Cd concentrations of 0.03 to 1500 ppt and Se concentrations of 0.02 to 1000 ppt. These concentrations were fixed to be lower than the QD concentrations classically used *in vivo* (ppb-ppm)^29^. QDs were also added, with the same target concentrations, to both the HNO_3_ 2% and NaNO_3_ 0.01 M matrices, used as control media. However, due to a high natural Zn concentration present in the plasma samples, an additional set of experiments was performed corresponding to the addition of 1000, 5000, 10000, 15000, 20000, 25000 and 30000 ppt of Zn to the QDs. All of the samples were triplicated to assess the experimental reproducibility.

After the QDs were added to these media, all of the samples were agitated and then acidified to obtain 2% of nitric acid in the samples (in the case of the blood plasma samples, 1.5% of hydrochloric acid was used as complete digestion was not reached using nitric acid), and then left overnight prior to dilution and analysis. This acidification step is representative of the final step used in many studies prior to ENP analysis^15,19^. Simple acidification was sufficient to completely dissolve the QDs, and therefore complete acid digestion with HF/HNO_3_ was not carried out. For the HNO_3_ 2% and NaNO_3_ 0.01 M matrices, no further dilution was needed prior to the HR-ICPMS analysis. For the rat plasma, DPBS solution, artificial urine and saliva samples, all of the samples were diluted 50-fold to ensure the best instrument performance and stability^30^.

### Quantitative analysis by ICP-MS

#### Calibration and internal standards

External standard solutions containing 1, 5, 10, 100, 500, 1000 and 5000 ppt of the total amount of Cd, Se and Zn were prepared in HNO_3_ 2% or 1.5% of hydrochloric acid (for blood plasma samples analysis). During the whole analysis, a solution containing 5 ppb of rhodium (^103^Rh) prepared in HNO_3_ 2% was used as the internal standard solution to correct for instrumental drift and mass bias.

#### Isotopic analyses by HR-ICP-MS

The isotopes of Cd (^106^Cd, ^108^Cd, ^110^Cd, ^111^Cd, ^112^Cd, ^113^Cd, ^114^Cd and ^116^Cd), Zn (^64^Zn, ^66^Zn, ^67^Zn, ^68^Zn and ^70^Zn) and Se (^74^Se, ^76^Se, ^77^Se, ^78^Se, ^80^Se, ^82^Se) were analyzed with HR-ICP-MS (ThermoScientific Element II). Isotopes ^105^Pd, ^115^In, ^118^Sn, ^60^Ni and ^72^Ge were also analyzed to correct for possible isobaric interferences. Each intensity used for the data processing corresponds to the average of 15 sets of three replicates which are used to obtain an internal reproducibility with a standard error less than 5%. Within each session, the reproducibility of the known Cd, Zn and Se standard solutions (Chem-Lab, Belgium) was checked at the beginning and end of each analysis sequence and yielded an average shift of 2.6% from the certified values. Most of the sub-procedural variation was found to be within the overall external reproducibility for the methods stated above, determined using the three experimental replicates.

#### Calculation of spiked QD concentrations

The contrast in the isotopic compositions between the multi-spiked QDs and the natural background forms the basis of the quantification of the QDs using HR-ICP-MS measurements. The calculation is adapted from Dybowska *et al*.^17^ and is detailed in the SI (Eq. S1 to S24).

## Results and Discussion

### Multi-spiked QD synthesis and characterization

**Figure 1:**
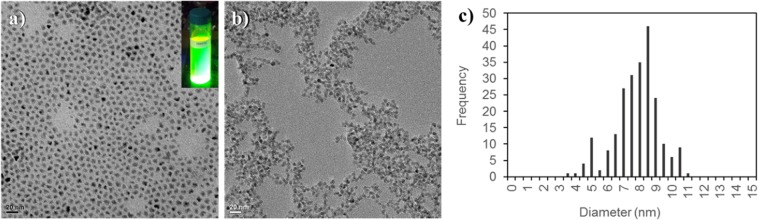
(**a**) TEM image of the multi-spiked CdSe/ZnS QDs dispersed in chloroform. (**b**) TEM image of the CdSe/ZnS TGA-coated QDs dispersed in Milli-Q® water. (**c**) Size distribution of the QDs estimated from (**a**)

The multi-spiked QDs obtained in this study emit a green color centered at 540 nm with a 40 nm-emission linewidth (Figure S1a, SI). This value is slightly larger than the one found from the non-spiked QDs (test run, Figure S1b, SI) synthesized using the usual zinc acetate precursor. This difference can be related to a slight quality degradation of the nanocrystals when compared to the non-spiked QDs from the test run, which is due to the use of unusual zinc oxide (ZnO) precursors during our synthesis. ZnO precursor dissolution was more difficult to achieve in comparison to the usual precursor (zinc acetate as mentioned in Bae’s report in 2008) yet it is feasible when the ZnO precursor powder is heated up to 250 °C for 10 minutes. Regardless of the difference found, the properties of our multi-spiked QDs are still comparable to that described by Bae. Our QDs are mostly round in shape and some are slightly oval as observed under TEM (Fig. 1a,b), with the estimated size distribution of the QDs of 7.6 ± 1.6 nm for *n* = 230 (Fig. 1c). The presence of Zn, Cd, and Se in the multi-spiked QDs was also verified by EDXS (Figure S2, SI).

As for the stock solution of the multi-spiked TGA-coated QDs in water used in this study, it has a pH of 10 and the concentrations of Zn, Cd and Se are 101.1 ± 1.0 ppm, 31.8 ± 0.3 ppm, and 19.2 ± 0.3 ppm, respectively. The solution also displays an enrichment in ^111^Cd, ^68^Zn and ^77^Se to 95.89, 99.11 and 98.95% (Figure S3, SI) as measured by HR-ICP-MS, consistent with the certified isotopic composition of the initial materials from ISOFLEX, yet slightly lower. Minor contamination in the chemicals used during the synthesis could be the cause, nevertheless, the QDs isotopic composition is in significant contrast to the natural isotopic abundances of ^111^Cd (12.80%), ^68^Zn (18.80%) and ^77^Se (7.64%).

### Analytical performance and limit of quantification of the QDs (QD-LOQ)

The R software (Bell Laboratories) was used for data processing and statistical analyses.

#### Precision assessment

**Figure 2:**
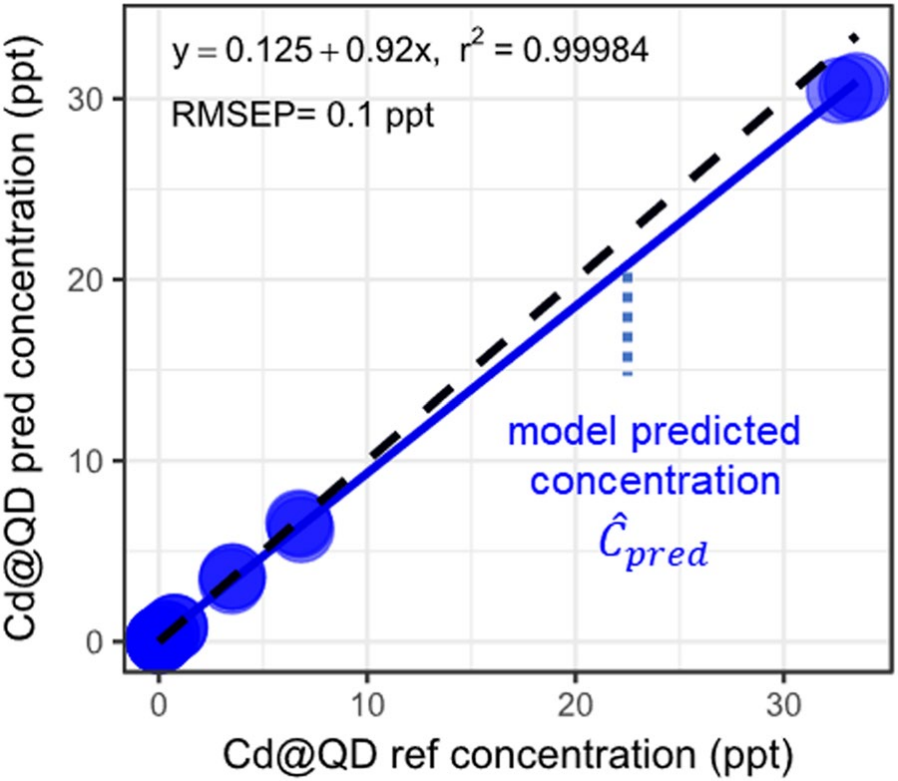
C_pred_ vs. C_ref_ recovery plots to estimate the concentration of the QDs based on the ^111^Cd tracer. The solid black line indicates the best linear fit and the dashed black line represents the ideal C_pred_ = C_ref_ recovery plot.

The recovery plot was built by representing the predicted QD concentrations (*i.e*. calculated using Eqs. S8, S16 and S24) as a function of the reference QD concentrations (*i.e*. the theoretical concentrations of the QDs added in the matrices). An example is shown in Fig. 2, using the DPBS matrix and based on the ^111^Cd tracer.

As discussed in a previous study^31^, the recovery rate of the analytical methods (*i.e*. the fraction of QDs detected among the QDs present in the sample), is obtained from the slope of the best linear regression model. The recovery rate is related to the bias defined as (1 – recovery) x 100. The Root Mean Square Error of Prediction (RMSEP, defined in Eq. 1) of the regression model plot allows to estimate the precision of the predicted concentration of the QDs.

1 $${RMSEP}=\sqrt{\frac{{\sum }^{}{({{C}}_{{pred},{i}}-{\hat{{C}}}_{{pred},{i}})}^{2}}{{n}}}{\rm{with}}\,{\rm{n}}\,{\rm{the}}\,{\rm{number}}\,{\rm{of}}\,{\rm{samples}}$$

#### QD-LOQ determination

The concentration for which the possibility of a false positive or negative falls below 0.1% was used to define the limit of quantification of the QDs (QD-LOQ). As an example, for a set of samples with a reference concentration C_0_, C_1_, C_2_, …, C_n_ where C_0_ corresponds to the “blank” samples, we determined the QD-LOQ by performing a t-test. This allowed the comparison between the mean value of the predicted concentrations of the blank samples and the mean value of the predicted concentrations of the samples using C_1_ as the reference. If the difference between the two means significant (at the 99.9% confidence level), the QD-LOQ was fixed equal to C_1_. If not, we carried out the t-test using the samples with C_2_ as the reference concentration. We then reproduced the process with increasing C_i_ concentrations until the t-value calculated exceed the critical t-value at the 99.9% confidence level. An overview of this method for the QD-LOQ determination from the recovery plot or its model is displayed in the SI (Figure S4).

Because the set of QD spiked samples covers decades from the ppt to the ppb levels, only a few experimental points were close enough to the QD-LOQ value. This implies that the QD-LOQ value determined from the series of t-tests only performed on the experimental data provide an overestimated value. Hence, a second series of t-tests was performed using the experimental blank samples and the model predicted values at intermediate concentrations, with $\widehat{{{C}}_{{i},{pred}}}$ as the mean and RMSEP as the standard deviation (SD). This estimation can also be considered conservative as it assigns overestimated SD to points at low reference concentrations since the RMSEP is calculated on the basis of data covering several decades. We then retained the lowest of the two QD-LOQ estimates. An overview of this method for the QD-LOQ determination from the recovery plot or its model is displayed in the SI (Figure S4).

The recovery plots of ^68^Zn, ^111^Cd and ^77^Se from the QDs are also presented in the SI (Figures S6 to S22). In addition, the SI (Table S4) summarize the biases of all of the recovery plots as well as the t-values for the various Cd, Zn and Se concentrations in the QDs calculated for all the matrices (Table S6,S8).

#### QD-LOQs and precision

**Figure 3:**
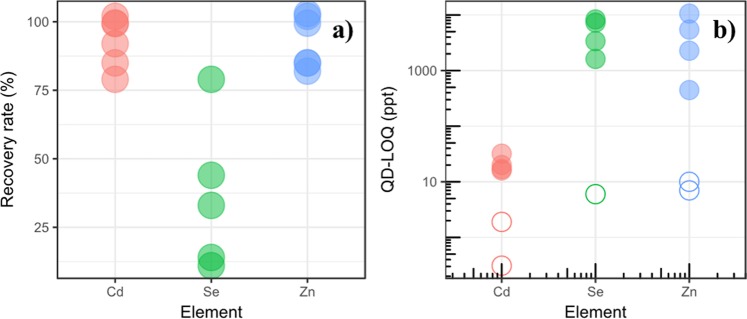
(**a**) Recovery rates grouped by tracer element for all matrices. (**b**) QD-LOQs grouped by tracer element for simple matrices (empty symbols) and complex matrices (filled symbols).

The recovery rates are displayed in Fig. 3 for each element. They are excellent for Cd and Zn at 93 ± 9.9% and 93 ± 10% on average for the studied biological matrices. Ifcompared to our previous work which focused in aquatic matrices, the recovery rates found for both elements were also excellent (*ca.* 97-99%), consistent with the values found in this study. The change in matrices (biological vs. aquatic matrices which contain differentbackground concentrations) does not seem to affect the recovery rates for Cd and Zn. However, the recovery rate is poor for Se, varying from 11 to 79%, indicating that the method is not sensitive to Se, which was also the case in our previous work (*ca.* 19%).

This is directly linked to the instrumental resolutions used for the analysis of the three different elements by ICP-MS. Whereas Cd isotopes were measured in low resolution (LR), since they are not strongly impacted by polyatomic or isobaric interferences, the Zn isotopes had to be measured in medium resolution (MR) to resolve polyatomic interferences with ^48^Ca^16^O^+^, ^32^S^16^O^18^O^+^, ^40^Ar^14^N_2_^+^, which resulted in count numbers approximately 90% below those typically measured in LR. Worst of all, Se had to be analyzed in high resolution (HR) to overcome polyatomic interferences mostly caused by argon (Ar) from the plasma, *e.g*. ^36^Ar^40^Ar^1^H^+^, 40Ar_2_^+^. The signal measured is then approximately 3% of the total signal measured in LR, which is related to the resolution slit width that defines the mass resolution, R (R_LR_ = 300, R_MR_ = 4000 and R_HR_ = 10000).

The ANOVA calculation performed through a model describing the relationship between the recovery rate and the ICP resolution as a linear function, allowed to confirm the significant impact of the resolution at the 99.9% confidence level (Tables S9,S12, models M1 and M2, SI). The count numbers were furthered lowered by the fact that the QD concentrations calculated from Se were lower on average than those for Cd and Zn, respectively. These results highlight the limitations of the spiking method for tracking NPs: for elements subjected to strong polyatomic interferences such as Se, it will hardly be possible to reach the ppb level when determining isotopically labelled nanoparticles concentration. This is proven by the recovery plot obtained for the blood plasma matrix which has a better recovery rate for Se (79%) when working at higher QD concentrations. However, some specific technical solutions such as hydride generation^32^ could be used to overcome this limitation.

#### QD-LOQs

**Figure 4:**
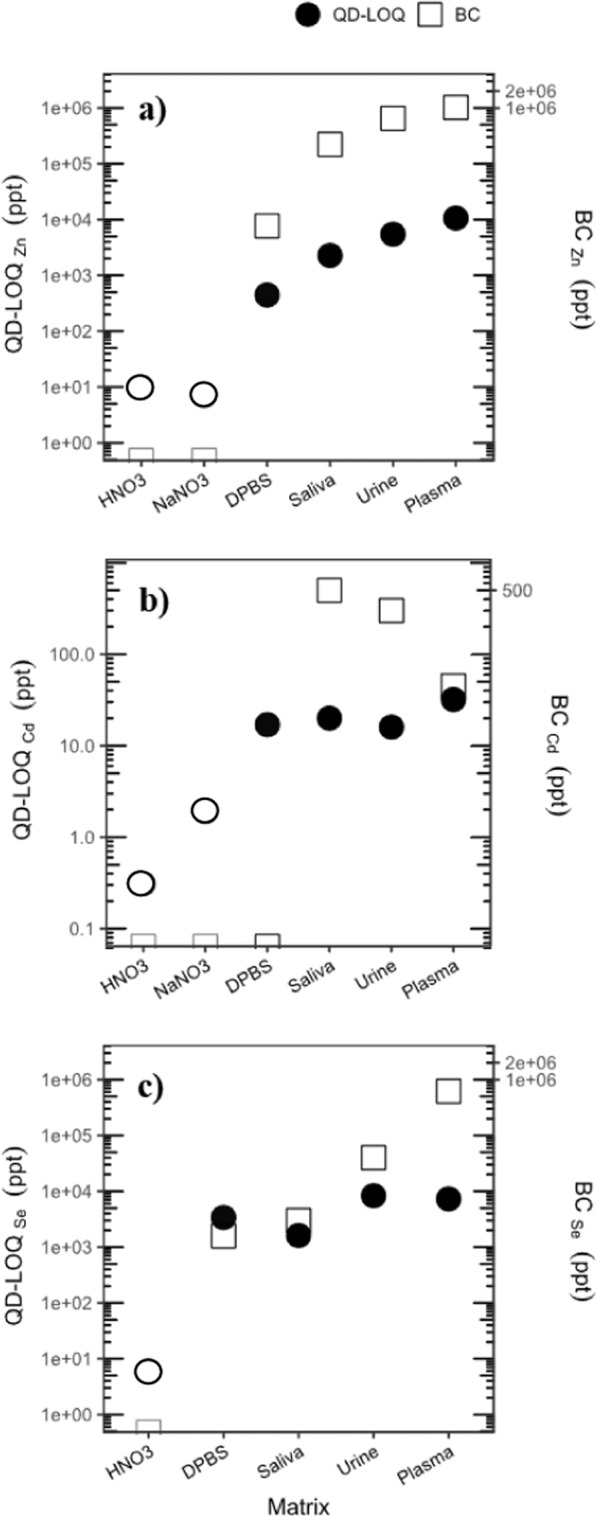
QD-LOQs grouped by tracer element for the biological matrices (●) compared with the background concentration (BC) in the matrix (□) for the studied elements: (**a**) Zn, (**b**) Cd and (**c**) Se. The black circles (●) represent the QD-LOQs found for 50-fold diluted samples, whereas the white circles (o) indicate the non-diluted samples.

Table 1 and Fig. 4 display the QD-LOQ values of the multi-spiked QDs grouped by tracer element (Zn, Cd, Se). In oversimplified media (HNO_3_ 2% and NaNO_3_ 0.01 N) where the background concentrations of Zn, Cd and Se are below the HR-ICP-MS LOQ values (30, 3 and 62 ppt for Zn, Cd and Se, respectively), the QD-LOQ values of Zn, Cd and Se are 10, 0.3 and 6 ppt in HNO_3_ 2% and 7 and 2 ppt for Zn and Cd in NaNO_3_ 0.01 N, respectively. These values demonstrate the strength and advantages of the isotopic labelling method at very low concentrations.

**Table 1:** Background concentration (BC), QD-LOQ, precision (RMSEP) and relative QD-LOQ (QD-RLOQ) in the biological matrices vs. simple matrices. The conventional LOQ values for the HR-ICP-MS used in the present study are: Zn 30 ppt, Cd 3 ppt and Se 60 ppt.

		HNO_3_ 2%	NaNO_3_ 0.01 M	Saliva	Urine	Plasma	DPBS
Zn	BC (ppt)	<LOQ_Zn_	<LOQ_Zn_	222 000	650 000	1020 000	7700
	**QD-LOQ (ppt)**	**10**	**7**	**2275**	**5459**	**10598**	**446**
	**Precision (ppt)**	**0.8**	**1**	**455**	**551**	**973**	**74**
	QD-RLOQ	—	—	1.0%	0.8%	1.0%	5.5%
Cd	BC (ppt)	<LOQ_Cd_	<LOQ_Cd_	500	300	45	<LOQ_Cd_
	**QD-LOQ (ppt)**	**0.31**	**1.9**	**20**	**16**	**32**	**17**
	**Precision (ppt)**	**0.11**	**0.3**	**7.8**	**7.2**	**11**	**6**
	QD-RLOQ	—	—	4%	5%	42%	—
Se	BC (ppt)	<LOQ_Se_	*n.a*.	3000	40 000	620 000	1580
	**QD-LOQ (ppt)**	**6**	***n.a****.*	**1618**	**8317**	**7291**	**3388**
	**Precision (ppt)**	**0.4**	***n.a****.*	**93**	**196**	**1009**	**55**
	QD-RLOQ	—	—	35%	17%	1%	68%

In contrast with the oversimplified media, the QD-LOQ values in the biological matrices significantly increase, varying from 446 to 10598 ppt for Zn, 16 to 32 ppt for Cd and 1618 to 8317 ppt for Se. More specifically, the respective QD-LOQs for Zn, Cd and Se are 2275, 20 and 1618 ppt in saliva, 5459, 16 and 8317 ppt in urine, 10598, 32 and 7291 ppt in plasma, and 446, 17 and 3388 in the DPBS growth medium.

In the following, the QD-LOQs for Se will not be discussed further given its poor recovery rates observed here, as discussed earlier. To summarize our results for the QD-LOQs, the mean values of the QD-LOQs for Cd and Zn are 15 and 3132 ppt, respectively. These values are lower than the typical concentrations used in ENP dissemination and toxicity studies (ppb-ppm level)^18,33–35^. Indeed, coherent to our previous findings in the case of aquatic matrices, the QD-LOQs found on average previously were also lower by about 3 to 6 orderof magnitudes than those in the aforementioned studies. Our results in this paper also demonstrate the significant added value of the isotopic labelling technique in order to model the behavior of ENPs in conditions as similar as possible to realistic biological media during toxicity studies.

#### Factors affecting the QD-LOQs

Three possible arguments can be used to explain the increase of the QD-LOQ values between the simplified media and the the biological matrices. First, the higher concentration of dissolved salts and organic matter in the biological matrices can affect the intensity readings in ICP-MS compared to the readings in ultrapure matrix like HNO_3_. The latter is the matrix in which the *I*(^A^*X*)*vs*.*C*_*A*_ calibration plots were acquired. This phenomenon is known as matrix effect. Therefore, the effect of ionic strength on the samples during the analyses in the instrument will be investigated, as a proxy of the concentration of dissolved salts.

Second, the biological samples contain high total dissolved solids (TDS) concentrations that may damage the instrument and drastically decrease its sensitivity. Consequently, these samples were diluted before the ICP-MS analysis to reach, at the very most, a TDS value of 2 g/L that is recommended for ICP-MS analysis. This inevitably resulted in lower tracer concentrations and lower count numbers. Hence, the effect of the dilution factor applied to each sample will be examined as a potential factor controlling the QD-LOQ.

Last, biological matrices contain higher BCs, *i.e*. increasing occurrences of the constitutive elements (namely Se, Cd and Zn occurring at natural isotopic abundances) of the quantum dots in the matrices, whereas HNO_3_ and NaNO_3_ matrices theoretically do not contain metals from the BC. For instance, the Zn background concentrations in the blank samples in both simplified media were below the LOQs, whereas they increase by up to 1020 ppb in complex matrices (here in plasma) *i.e*. 34 times higher than the upper limit of the QD concentration ranges investigated in the present study. In other words, this results in a dilution of the characteristic isotopic fingerprint of the multi-spiked QDs when the BC contribution with natural isotopic abundances is large. The effect of the BC on the tracer element in each sample will be tested.

In the following paragraphs, the QD-LOQ values for Se will not be discussed due to its poor recovery rates: because Se has to be measured in HR, this induces a loss of sensitivity on ICP-MS measurement.

First, the acidification at 2% of HNO_3_ (or 1.5% of HCl for the plasma samples) prior to analysis controls and levels the ionic strength of the solutions analyzed in ICP-MS (not shown here) whatever the various matrices analyzed. Therefore, matrix effects cannot account for the increase in the QD-LOQ values observed when moving from simple media to complex aquatic matrices. Second, for both Cd and Zn, the lowest QD-LOQs are the ones determined in the simple matrices that did not require dilution prior to analysis; thus, the dilution factor seems to have a significant impact on the QD-LOQ values since all of the biological samples were diluted 50-fold. Last, for Zn, the QD-LOQs seem to be correlated with the BC and ANOVA calculations were performed to confirm these findings.

The modelling of the whole set of QD-LOQs for both Zn and Cd revealed that the BCs were significant at the 99.9% confidence level (Table S13, model M3, SI). Nevertheless, the QD-LOQs calculated with Zn were found to only depend on the BC (Table S14,S15, model M4, Figure S23, SI), while those based on Cd did not depend on the BC but were correlated with the dilution factor (Table S16,S17, model M5, Figure S24, SI). The reported r² value obtained = using model M4 (log QD-LOQ_Zn_ *vs*. log BC) is 0.9957, suggesting that, for the studied biological matrices, the BC_Zn_ values have an influence on the QD-LOQ_Zn_ values. This shows that when the concentrations of natural Zn in the biological matrices are higher, the QD-LOQs values will also be higher. For Cd, the reported r² value obtained using model M5 (QD-LOQ_Cd_ *vs*. dilution factor) is 0.8237, showing a correlation between QD-LOQ_Cd_ and the dilution factor.

Since these studied factors (analysis resolution, dilution factor, or BC in the matrices) have been shown to affect the QD-LOQs differently for each studied element (Zn, Cd, Se), they should be taken into account when fixing the concentration of spiked QDs (or spiked ENPs containing Zn/Cd/Se) to be used in ENP fate and toxicity studies. These factors should be studied in other types of matrices as well, for example as carried out in aquatic matrices in our previous study.

#### Relative QD-LOQ (QD-RLOQ)

The relative QD-LOQ (QD-RLOQ) is used in order to assess accurately the spiking method efficiency by considering the BC in the matrix. It is calculated by dividing the QD-LOQ by the sum of the total natural element concentration (background concentration) already present in the matrix and the QD-LOQ (Eq. 2). If compared to what has been done in our previous work, the term BC in this equation here has replaced the term GBC (geochemical background concentrations in aquatic matrices).

2 $${QD}\,{RLOQ}=\frac{{QD}\,{LOQ}}{{QD}\,{LOQ}+{BC}}\,\ast 100$$

Table 1 summarizes the QD-LOQs and relative QD-RLOQs (QD-RLOQs) for Se, Cd and Zn in all of the studied matrices. In the case of Zn, although the QD-LOQs seem to be much higher than those found for the simplified media, they indicate that Zn from the multi-spiked QDs can be detected and quantified in saliva, urine and plasma as low as 1.0, 0.8 and 1.0%, respectively, of the medium Zn background concentration (BC_Zn_). In the case of the DPBS growth medium, this percentage (QD RLOQ) was found to be 5.5%. The 50-fold dilution factor does not have significant effect on the QD-RLOQ_Zn_ values because the total Zn concentration in the matrices was generally higher than Cd concentration.

For Cd, the QD-RLOQs in saliva, urine and plasma are 4, 5 and 42%, respectively. The QD-RLOQ_Cd_ value in DPBS was not calculated because the level of BC_Cd_ detected was below the HR-ICP-MS LOQ ( < 3 ppt). Since the QD-LOQs for Cd are not influenced by the background level of Cd, as discussed earlier, this simultaneously explains the irregular QD-RLOQ_Cd_ values between all of the matrices (compared to the QD-RLOQ_Zn_). The effect of the dilution factor can be seen in the higher QD-RLOQ values. The BC_Cd_ level in the blood plasma was 45 ppt compared with 500 ppt in saliva, making it difficult to detect Cd after the 50-fold dilution factor, resulting in a higher QD-RLOQ_Cd_ value (42%). As the total Cd concentration in the diluted samples was low, this induces a higher QD-RLOQ_Cd_ than that observed for Zn. This dilution was necessary in order to obtain a better performance and better stability during the analysis^30^, as the TDS of saliva, urine and DPBS are 6.4, 39 and 12 g L^−1^. In addition, the concentrations of Zn in saliva, urine and plasma, as well as that of Se in urine and plasma were too high (ppm level), which can saturate and damage the ICP-MS detector.

For Se, the QD-RLOQs are 35, 17, 1 and 68% in saliva, urine, plasma and DPBS, respectively. To briefly discuss the Se data, the difficulty detecting Se after the 50-fold dilution factor results in a higher QD-RLOQ_Se_ value for DPBS compared to the plasma matrix (68% *vs*. 1%) since the initial concentration of Se (BC) in the DPBS was much lower compared to that of plasma (1580 *vs*. 620 × 10^3^ ppt).

Lastly, for a brief comparison overview of our both present and previous studies, the QD-RLOQs of Zn, Cd, and Se in biological matrices vary from 0.8–5.5%, 4–42%, and 1.0–68%, respectively, meanwhile in aquatic matrices they range from 1.0–7.7%, 29–49%, and 11–80%, respectively (Figure S25). We suggest that these values could help in estimating the lowest concentration to be used in future studies based on the background level in the medium.

#### Perspectives: spiked QDs as an alternative to optical properties when working at relevant and low concentrations

The QD-RLOQs obtained during our experimental work can be applied to the detection and quantification of Zn/Cd/Se-spiked ENPs by HR-ICP-MS in most biological matrices containing Zn/Cd/Se and may be useful in toxicological studies that aim to work at relevant NP concentrations. For instance, when the optical properties of QDs in a biological medium are no longer visible at low concentrations, the isotopic labelling technique can be used as an alternative method to detect and quantify them, especially when tracing their behavior (*e.g*. dissolution, aggregation) for improved understanding in nanotoxicology studies. Multi-spiked QDs could be the solution to overcome analytical barriers such as constitutive elements of the QDs that are endogenously present in biological matrices. If the concentrations of naturally present Zn in the biological media are known beforehand, the QD-RLOQ could be used first to accurately determine the QD-LOQ of the ENPs, as the lowest ENP concentrations that should be used in the experiments. As suggested in our previous study, the RLOQ values defined in this study can also be used to determine the concentration of ENPs containing Zn/Cd/Se to be used for toxicity/fate/behavior experiments using the isotopic labelling technique. By referring to the QD-LOQs estimated in this study, future studies related to ENPs toxicity, fate, and behavior could be carried out at realistic concentrations using isotopically labelled ENPs. We suggest that future experimental works should be done at relevant concentrations to improve our understanding of ENPs toxicity, fate, and behavior. This could be performed by combining the methodology developed in the present study and additional techniques such as chronopotentiometry, ultrafiltration and HR-ICP-MS.

**Figure 5:**
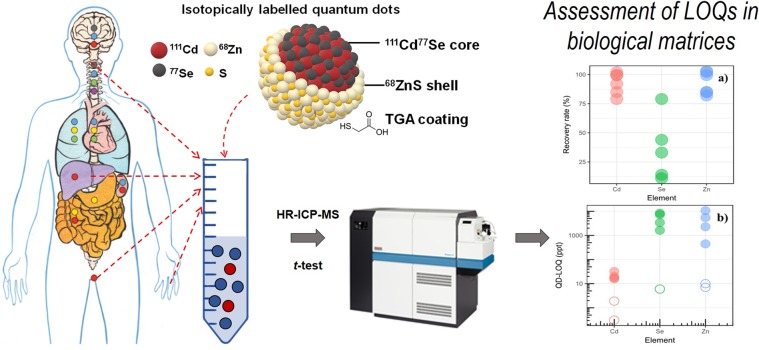
Global concept of the study.

In summary, our methodology which combines both HR-ICP-MS and chemometric analysis (Fig. 5) allowed to accurately determine the limits of quantification of isotopically labelled CdSe/ZnS quantum dots (QD-LOQs) in biological matrices. The quantification levels reported in this study range from 16 to 32 ppt for Cd, 446 to 10598 ppt for Zn and 1618 to 8317 ppt for Se. Based on our findings, isotopic labelling of ENPs coupled with HR-ICP-MS will enable researchers to work at ENP concentrations lower than the usual range of concentrations used in ENP studies related to their fate, behavior, and toxicity (e.g. QDs at 60–3600 ppb)^33,34^. In addition, this method does not require the use of specific ENP characteristics (e.g. fluorescence, optical emission) such as QDs. The method is more sensitive if compared to methods depending on photoluminescence which require the use of much higher concentrations for a precise measurement^36^.

## Supplementary information

Supporting Information.
