# Supplementary material for: Tracing multi-isotopically labelled CdSe/ZnS quantum dots in biological media
Source: Sci Rep. 2020 Feb 18;10:2866. doi: 10.1038/s41598-020-59206-w (PMC7028726; doi:10.1038/s41598-020-59206-w)
Supplement: Supplementary file 1 — Supporting Information. [file 41598_2020_59206_MOESM1_ESM.pdf]

SUPPORTING INFORMATION

Tracing multi-isotopically labelled CdSe/ZnS quantum dots in biological environments: an assessment of the method's limitations

N. Izyan Supiandi[a], G. Charron[b], M. Tharaud[a], M. F. Benedetti[a], and Y. Sivry[a]*

[a]Institut de Physique du Globe de Paris, Sorbonne Paris Cité, Univ. Paris Diderot, UMR 7154, CNRS, F-75005 Paris, France; [b] Laboratoire Matière et Systèmes Complexes (MSC), Université Paris Diderot, 75013 Paris, France

*Corresponding author: sivry@ipgp.fr

List of contents

Synthesis of multi-isotopically labelled CdSe/ZnS quantum dots..3

Preparation of water-soluble QDs: Thioglycolic Acid (TGA)-coated QDs 4

Acid digestion protocol..5

Composition of biological matrices 6

Calculation of spiked QDs concentrations..9

Characterization of synthesized QDs..15

Overview of the methodology 17

Daily calibration of ICP signals as a function of isotope concentration – determination of the standard error on concentration (SE) and of the limit of quantification (LOQ) 18

Recovery plots: Bias 31

t table 32

Statistic *t* values calculated in all matrices 33

ANOVA Test Tables...35

QD-RLOQs comparison overview 39

Synthesis of multi-isotopically labelled CdSe/ZnS quantum dots

Briefly, 0.4 mmol of CdO, 4 mmol ZnO, 23.88 mmol of oleic acid and 23 mL octadecene were placed in a 100-mL round flask with 20 mg of succinic anhydride. The mixture was degassed under 10 mbar and then heated to 150°C and the montage was kept under 10 mbar at this temperature for 20 min. It was then filled with N_2 and then heated to 180°C and then 250°C to dissolve all the ZnO powder and the temperature was then increased slowly to 310°C. In the meantime, (before the montage reached 310°C), 0.4 mmol selenium and 4 mmol sulphur were inserted into a 20-mL vial equipped with a rubber septum. The vial was purged with N_2 from the montage using a double tip needle. Next, 3 mL of trioctylphosphine were taken out using a 5-mL syringe under N_2 flux and inserted into the vial still under N_2 flux. The vial was sonicated until dissolution of the S and Se powders. When the temperature of the reaction flask reached 310°C, a syringe was purged with N_2 and then used to take out the TOP/S/Se mixture. This chalcogenide mixture was quickly injected into the reaction flask under vigorous stirring. Immediately after injection, the target temperature was then quickly set to 300°C and the flask was kept at this temperature for 15 min. After the 15-min growth time, the flask was cooled down to room temperature. The QDs were precipitated with 100 mL of acetone by centrifugation at 6000 rpm and then purified by redispersion-precipitation cycles twice with 90 mL acetone and once with 40 mL methanol.

Preparation of water-soluble QDs: Thioglycolic Acid (TGA)-coated QDs

Water-soluble QDs were prepared by replacing the oleic acid attached to the surface of the QDs with thioglycolic acid (TGA). In a 100-mL round flask equipped with a refrigerant and a water bath, 460 µL of the multi-spiked QDs stock solution previously obtained, 10 mL of chloroform and 1.6 mL of thioglycolic acid (TGA) were added and sonicated. The reaction mixture was stirred and heated under reflux in water bath for 2 hours and then cooled to room temperature. The TGA-coated QDs were extracted by centrifugation at 6000 rpm, and then purified by redispersion-precipitation cycles twice with 10 mL chloroform and once with 10 mL acetone. Finally, the TGA-coated QDs were dispersed in pH 8 borate buffer and purified using Centricon® Centrifugal Filter Units (30kD MWCO) by exchanging the solvent once with a fresh portion of pH 8 borate buffer and 4 times with ultrapure water adjusted at pH 10. The final solution had a volume of 10 mL.

Acid digestion protocol

100 µL of TGA-coated QDs stock solution was transferred into a 60-mL pre-cleaned Savillex perfluoroalkoxy alkane (PFA) vial. 2 mL of suprapur hydrofluoric acid (HF, 27N) and 1 mL of bidistilled nitric acid (HNO_3 16N) were added into the vial. After putting on the vial's cap, it was then kept at 120°C during 24h.

After the 24-h digestion, the sample was evaporated to dryness at 80°C. The vial was cooled down to room temperature, and all droplets on the vial's wall were retrieve carefully. Afterwards, 0.5 mL of bidistilled HNO_3 (16N) was added into the vials and closed vials was kept at 120°C for 2h. After cooled down, the sample was retrieved with 9.5 mL of Milli-Q water during a transfer to a pre-cleaned 15-mL polypropylene (PP) tube.

Composition of biological matrices

Table S1. Artificial urine recipe

Name	Chemical formula	Concentration (g/L)
Sodium chloride	$NaCl$	11.6
Diammonium hydrogen phosphate	$(NH_4)_2 HPO_4$	2.0
Urea	$H_2N CO NH_2$	18.0
Creatinine	$C_4H_7N_3O$	1.0
Uric acid	$C_5H_4N_4O_3$	0.25
Tartrazine	$C_{16}H_9N_4Na_3O_9S_2$	0.1
Monopotassium phosphate	$K H_2PO_4$	0.7
Disodium phosphate dodecahydrate	$Na_2HPO_4.12H_2O$	5.3
pH 7.4		
		Measured concentration (ng/L)
Zinc	Zn	650.10^3
Cadmium	Cd	300
Selenium	Se	400.10^3

Table S2. Artificial saliva recipe

Name	Chemical formula	Concentration (g/L)
Sodium chloride	$NaCl$	1.594
Ammonium nitrate	$NH_4\ NO_3$	0.328
Potassium phosphate	$K\ H_2PO_4$	0.636
Potassium chloride	KCl	0.202
Potassium citrate monohydrate	$K_3C_6H_5O_7.H_2O$	0.308
Uric acid sodium salt	$C_5H_3N_4O_3\ Na$	0.021
Urea	$H_2N\ CO\ NH_2$	0.198
Lactic acid sodium salt	$CH_3CH(OH)CO_2\ Na$	0.146
Mucin from porcine stomach		3.000
pH 6.8		
		Measured concentration (ng/L)
Zinc	Zn	222.10^3
Cadmium	Cd	500
Selenium	Se	3000

Table S3. DPBS growth medium recipe

Name	Chemical formula	Concentration (g/L)
Sodium chloride	$NaCl$	8.0
Potassium chloride	KCl	0.2
Disodium phosphate bihydrate	$Na_2HPO_4.2H_2O$	1.44
Monopotassium phosphate	KH_2PO_4	0.2
Calcium chloride bihydrate	$CaCl_2.2H_2O$	0.13
Magnesium chloride hexahydrate	$MgCl_2.6H_2O$	0.1
pH 7.2 - 7.4		
		Measured concentration (ng/L)
Zinc	Zn	7700
Cadmium	Cd	< LOQ
Selenium	Se	1580

Calculation of spiked QDs concentrations

Zinc

The contrast in isotopic compositions between the multi-spiked QDs and the natural background forms the basis of the quantification of the QDs from HR-ICP-MS measurements. For instance, in the case of Zn, the concentration of ^{68}Zn in a biological sample containing spiked QDs, is the sum of the contributions of the QDs stock solution and of the matrix (Eq. S1). The same is true for the concentration of ^{66}Zn (Eq. S2).

$$C_{68}^{sample} = C_{68}^{matrix} + C_{68}^{QD} \qquad \text{(Eq. S1)}$$

$$C_{66}^{sample} = C_{66}^{matrix} + C_{66}^{QD} \qquad \text{(Eq. S2)}$$

However, as the multi-spiked QDs feature an almost pure ^{68}Zn isotopic composition, the ^{66}Zn contribution from the QDs can be neglected (Eq. S3). The total Zn contribution coming from the QDs, all isotopes included, therefore reduces to the ^{68}Zn contribution (Eq. S4).

$$C_{66}^{QD} \approx 0 \qquad \text{(Eq. S3)}$$

$$C_{total}^{QD} \approx C_{68}^{QD} \qquad \text{(Eq. S4)}$$

Eq. S1 & S2 then reorganize as Eq. S5 & S6, the latter expressing the concentration of Zn stemming from the QDs as a function of the ^{68}Zn concentration in the complex sample and the contribution of the matrix to that concentration.

$$C_{66}^{sample} \approx C_{66}^{matrix} \qquad \text{(Eq. S5)}$$

$$C_{total}^{QD} \approx C_{68}^{sample} - C_{68}^{matrix} \qquad \text{(Eq. S6)}$$

The ^{68}Zn contribution of the matrix can be inferred from its ^{66}Zn contribution given the natural abundances of ^{66}Zn and ^{68}Zn (Eq. S7).

$$C_{68}^{matrix} = C_{66}^{matrix} \times \frac{A_{68}^{natural}}{A_{66}^{natural}} \qquad (Eq.\ S7)$$

Combining Eq. S5, S6 and S7, the concentration of Zn stemming from the QDs in the complex sample expresses as a function of C_{68}^{sample} and C_{66}^{sample} (Eq. S8), which can be measured by comparison to calibration plots acquired using standards of natural isotopic compositions (Figure S5).

$$C_{total}^{QD} \approx C_{68}^{sample} - C_{66}^{sample} \times \frac{A_{68}^{natural}}{A_{66}^{natural}} \qquad (Eq.\ S8)$$

A similar calculation scheme was used to infer the concentrations of QDs in terms of Cd (based on ^{111}Cd and ^{110}Cd concentrations) and in terms of Se (based on ^{77}Se and ^{76}Se concentrations), as detailed in the next pages.

N.B. The concentrations in ^{66}Zn, ^{68}Zn, ^{76}Se, ^{77}Se, ^{110}Cd and ^{111}Cd were calculated by comparison to $I(\,^{A}X)\ vs.\ C(\,^{A}X)$ calibration plots of each isotope. The details on the daily determination of the conventional HR-ICP-MS limit of quantification (LOQ) are also presented in SI later (Figure S5).

Cadmium

The contrast in isotopic compositions between the multi-spiked QDs and the natural background forms the basis of the quantification of the QDs from HRICP-MS measurements. For instance, in the case of Cd, the concentration of ^{111}Cd in a biological sample containing spiked QDs is the sum of the contributions of the QDs stock solution and of the matrix (Eq. S9). The same is true for the concentration of ^{110}Cd (Eq. S10).

$$C_{111}^{sample} = C_{111}^{matrix} + C_{111}^{QD} \qquad \text{(Eq. S9)}$$

$$C_{110}^{sample} = C_{110}^{matrix} + C_{110}^{QD} \qquad \text{(Eq. S10)}$$

However, as the multi-spiked QDs feature an almost pure ^{111}Cd isotopic composition, the ^{110}Cd contribution from the QDs can be neglected (Eq. S11). The total Cd contribution coming from the QDs, all isotopes confounded, therefore reduces to the ^{111}Cd contribution (Eq. S12).

$$C_{110}^{QD} \approx 0 \qquad \text{(Eq. 11)}$$

$$C_{total}^{QD} \approx C_{111}^{QD} \qquad \text{(Eq. 12)}$$

Eq. S9 & S10 then reorganize as Eq. S13 & S14, the latter expressing the concentration of Cd stemming from the QDs as a function of the ^{111}Cd concentration in the complex sample and the contribution of the matrix to that concentration.

$$C_{110}^{sample} \approx C_{110}^{matrix} \qquad \text{(Eq. S13)}$$

$$C_{total}^{QD} \approx C_{111}^{sample} - C_{110}^{matrix} \qquad \text{(Eq. S14)}$$

The ^{111}Cd contribution of the matrix can be inferred from its ^{110}Cd contribution given the natural abundances of ^{110}Cd and ^{111}Cd (Eq. S15).

$$C_{111}^{matrix} = C_{110}^{matrix} \times \frac{A_{111}^{natural}}{A_{110}^{natural}} \qquad \text{(Eq. S15)}$$

Combining Eq. S13, S14 and S15, the concentration of Cd stemming from the QDs in the complex sample expresses as a function of C_{111}^{sample} and C_{110}^{sample} (Eq. S16), which can be measured by comparison to calibration plots acquired using standards of natural isotopic compositions.

$$C_{total}^{QD} \approx C_{111}^{sample} - C_{110}^{sample} \times \frac{A_{111}^{natural}}{A_{110}^{natural}} \qquad \textit{(Eq. S16)}$$

Selenium

The contrast in isotopic compositions between the multi-spiked QDs and the natural background forms the basis of the quantification of the QDs from HRICP-MS measurements. For instance, in the case of Se, the concentration of ^{77}Se in a biological sample containing spiked QDs is the sum of the contributions of the QDs stock solution and of the matrix (Eq. S17). The same is true for the concentration of ^{76}Se (Eq. S18).

$$C_{77}^{sample} = C_{77}^{matrix} + C_{77}^{QD} \qquad \textit{(Eq. S17)}$$

$$C_{76}^{sample} = C_{76}^{matrix} + C_{76}^{QD} \qquad \textit{(Eq. S18)}$$

However, as the multi-spiked QDs feature an almost pure ^{77}Se isotopic composition, the ^{76}Se contribution from the QDs can be neglected (Eq. S19). The total Se contribution coming from the QDs, all isotopes confounded, therefore reduces to the ^{77}Se contribution (Eq. S20).

$$C_{76}^{QD} \approx 0 \qquad \textit{(Eq. S19)}$$

$$C_{total}^{QD} \approx C_{77}^{QD} \qquad \textit{(Eq. S20)}$$

Eq. S17 & S18 then reorganize as Eq. S21 & S22, the latter expressing the concentration of Se stemming from the QDs as a function of the ^{77}Se concentration in the complex sample and the contribution of the matrix to that concentration.

$$C_{76}^{sample} \approx C_{76}^{matrix} \qquad \textit{(Eq. S21)}$$

$$C_{total}^{QD} \approx C_{77}^{sample} - C_{77}^{matrix} \qquad \textit{(Eq. S22)}$$

The ^{77}Se contribution of the matrix can be inferred from its ^{76}Se contribution given the natural abundances of ^{76}Se and ^{77}Se (Eq. S23).

$$C_{77}^{matrix} = C_{76}^{matrix} \times \frac{A_{77}^{natural}}{A_{76}^{natural}} \qquad \textit{(Eq. S23)}$$

Combining Eq. S21, S22 and S23, the concentration of Se stemming from the QDs in the complex sample expresses as a function of C_{77}^{sample} and C_{76}^{sample} (Eq. S24), which can be measured by comparison to calibration plots acquired using standards of natural isotopic compositions.

$$C_{total}^{QD} \approx C_{77}^{sample} - C_{76}^{sample} \times \frac{A_{77}^{natural}}{A_{76}^{natural}} \qquad \textit{(Eq. S24)}$$

Characterization of synthesized QDs

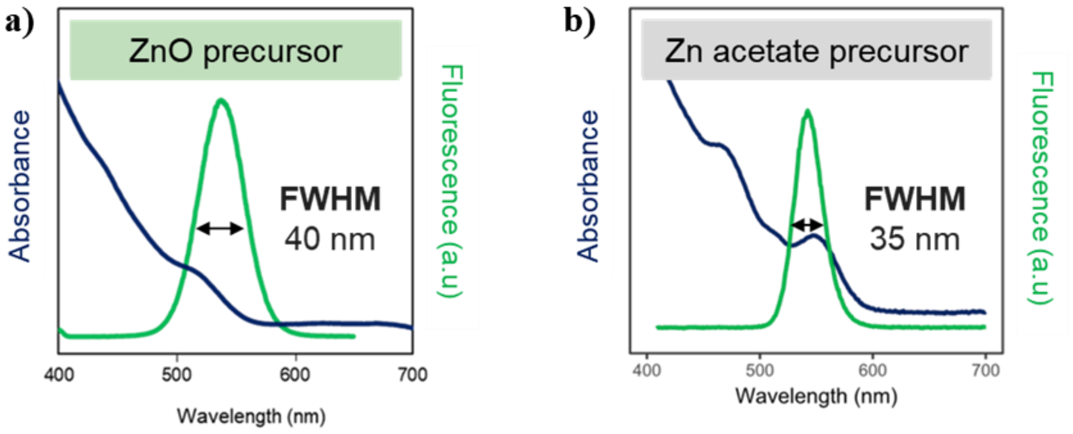

Figure S1. The blue line represents the UV-vis absorption spectrum of the multi-spiked quantum dots and the green line is the corresponding fluorescence emission spectrum under 400 nm excitation. The Full Width at Half Maximum (FWHM) *i.e.* emission linewidth, is presented by the black arrow. a) non-spiked QDs synthesized using the original protocol from Bae's report *i.e.* using zinc acetate precursor. b) multi-spiked QDs using ZnO precursor during synthesis.

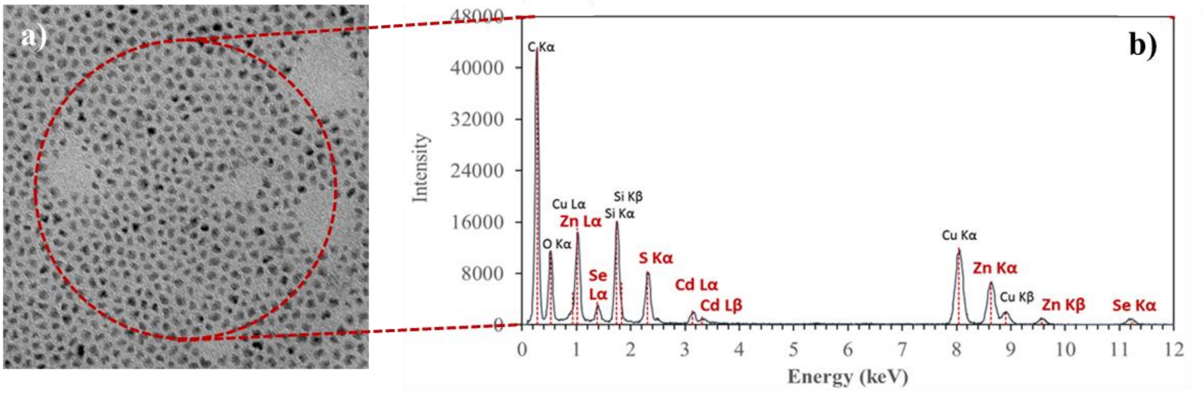

Figure S2. a) TEM image of QDs dispersed in chloroform. b) EDXS spectrum confirming the presence of Zn, Cd, and Se in the QDs.

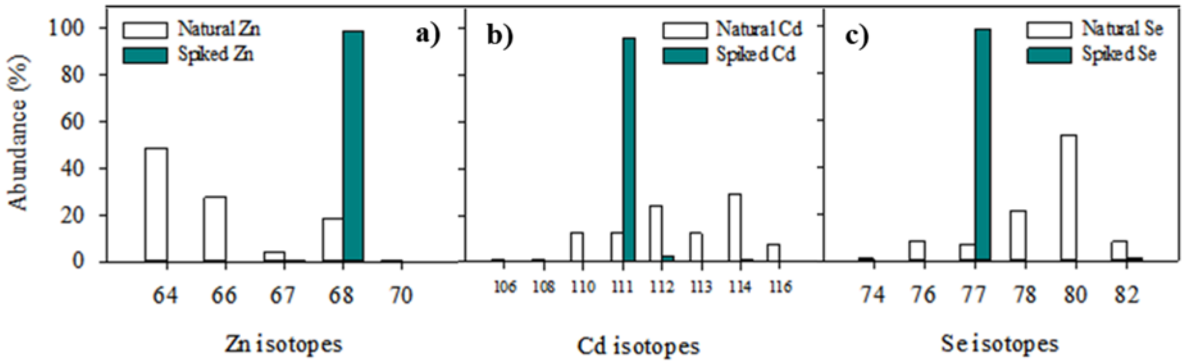

Figure S3. Comparison of natural and modified isotopic abundance of QDs for tracer elements. a) Zn, b) Cd, and c) Se.

Overview of the methodology

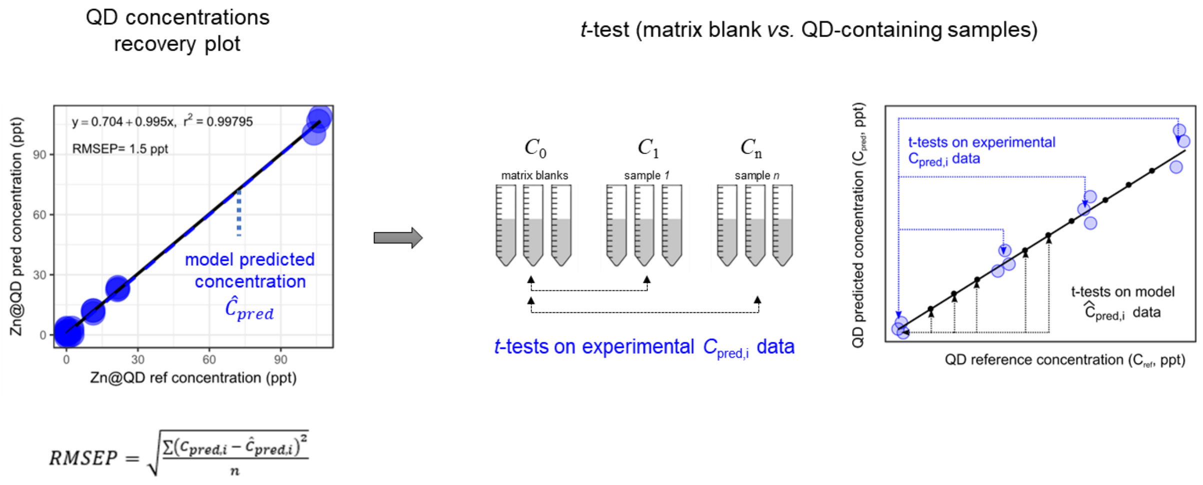

Figure S4. Overview of methodology applied to estimate the QD-LOQ from the recovery plot or its model – consist of two main steps: i) build the QDs concentrations recovery plots, and ii) run *t*-test on both experimental and model data.

Daily calibration of ICP signals as a function of isotope concentration – determination of the standard error on concentration (SE) and of the limit of quantification (LOQ)

Every day, new calibration plots were acquired by analyzing in ICP-MS elemental standards of each element having natural isotopic abundances. For a given tracer element, the signal intensity corresponding to isotope ${}^{A}X$ was plotted against the concentration of that isotope and the process was repeated for the second isotope of interest, as exemplified in Figure S5 in the case of Zn on the day the HNO_3 matrix was analysed.

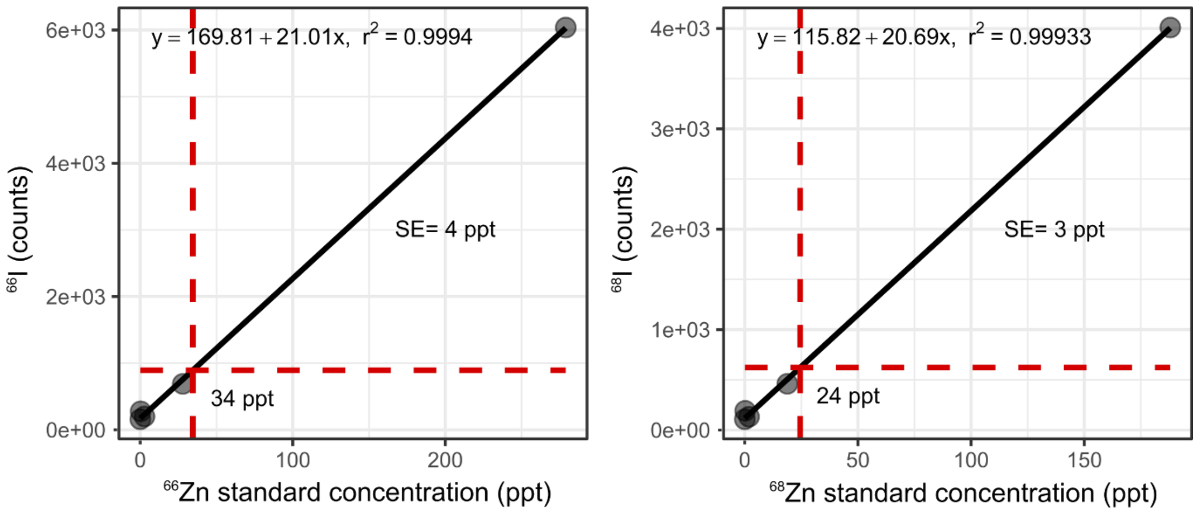

Figure S5. a) and b) $I({}^{A}Zn)\ vs.\ C({}^{A}Zn)$ calibration plots for ${}^{66}Zn$ and ${}^{68}Zn$. The horizontal and vertical dashed red lines indicate the LOQ and corresponding threshold intensity, respectively.

To obtain these calibration plots, the concentrations of each isotopes in the elemental standards were calculated as the products of the total concentration of the element in the standard and the natural abundances of the isotopes, as expressed in Eq. S25 and S26 in the case of the Zn tracer (for which $A^{66} = 27.9$ % and $A^{68} = 18.8$ %).

$$C(^{66}Zn) = C^{ref}_{Zn\,tot} \times A^{66} \qquad (Eq.\ S9)$$

$$C(^{68}Zn) = C^{ref}_{Zn\,tot} \times A^{68} \qquad (Eq.\ S10)$$

$$\hat{I}(^{A}X) = a + bC(^{A}X). \qquad (Eq.\ S11)$$

$$I(^{A}X)_{nuc-LOQ} = I(^{A}X)_{blank} + 10sd_{blank} \qquad (Eq.\ S12)$$

$$s_{blank} \approx s_{I/C} = \sqrt{\frac{\sum_{i=1}^{n}(I_i-\hat{I}_i)^2}{n-2}}$$, where n is the number of data points (*Eq. S13*)

$$s_C \approx \frac{1}{n}\sum_{i=1}^{n} s_{C_i} = \frac{1}{n}\sum_{i=1}^{n}\left(\frac{s_{I/C}}{b}\sqrt{1+\frac{1}{n}+\frac{(I_i-\bar{I})^2}{b^2\sum_{i=1}^{n}(C_i-\bar{C})^2}}\right)$$, where $\bar{C}$ and $\bar{I}$ are the mean values of concentration and intensity over the calibration range, respectively. (*Eq. S14*)

Linear regressions were applied to extract the calibration models (Eq. S27) which later afforded the estimation of concentrations in QD samples. The analytical performances of these calibration models were expressed as the limit of quantification for the given isotope (conventional LOQ) and the random error on the estimation of concentration from that model.

For all isotopes, the conventional LOQ was chosen as the concentration giving rise to a signal intensity equal to that of the blank plus 10 standard deviations of the blank (a choice that lowers the risk of false positive or false negative to about 10^{-4} %, Eq. S28). An estimate of the standard deviation of the blank was given by the random error in the intensity direction $s_{I/C}$ (so-called "random error in the y direction" in textbooks), as expressed in Eq. S29.

The standard error on the estimation of concentration using the calibration model was taken as the mean value of the standard errors in the concentration direction (so-called "random error in the x direction" in textbooks) on all data points, as expressed in Eq. S30.

HR-ICP-MS calibration and spiked QDs concentration recovery plots

HNO_3 2% matrix

Zinc

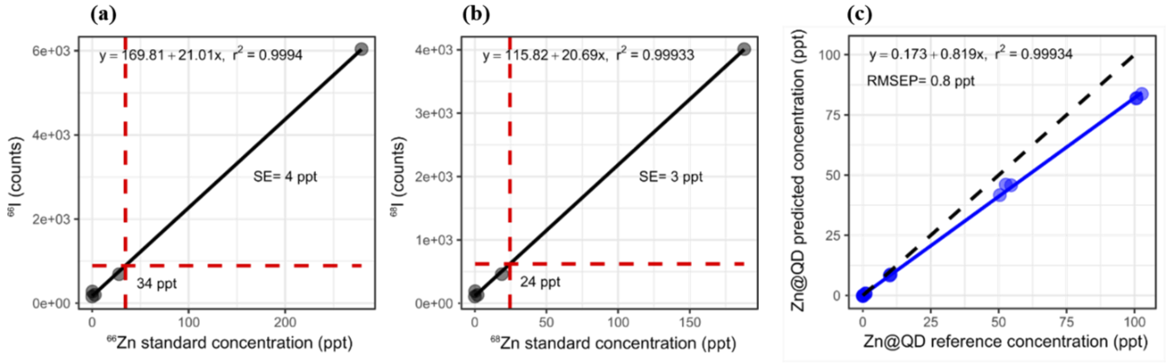

Figure S6. a) and b) Calibration plot of ^{66}Zn and ^{68}Zn. c) Recovery plot of multi-spiked QDs in ^{68}Zn in the HNO_3 2% matrix. The blue line indicates the best fit of the experimental data while the black dashed line is the ideal non-biased recovery plot ($C^{predicted}_{Zn@QD} = C^{reference}_{Zn@QD}$).

The recovery plot of Zn of the multi-spiked QDs in the HNO_3 2% features an 82% recovery rate, which indicates a negative bias of 18%. The precision on the determination of the QDs concentration was 0.8 ppt, obtained from the RMSEP of the regression line on the range 0-100 ppt of Zn coming from the QDs. The bias of all the recovery plots of spiked QDs concentration are summarized in Table S4.

Cadmium

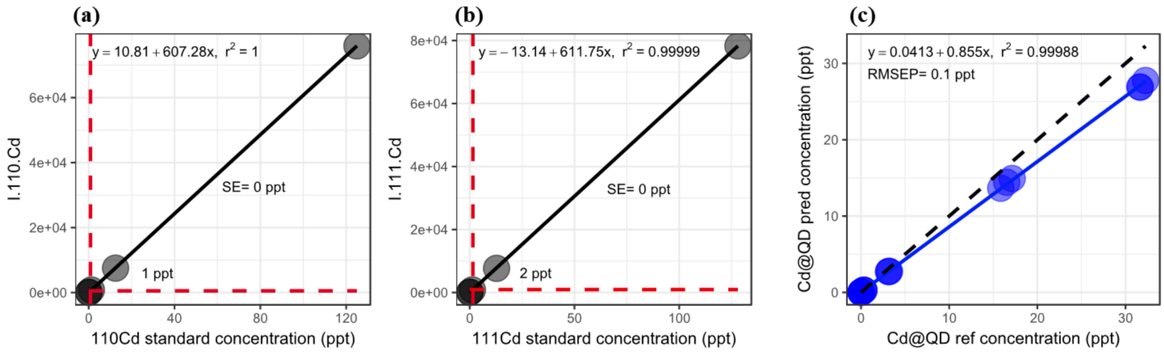

Figure S7. a) and b) Calibration plot of ^{110}Cd and ^{111}Cd. c) Recovery plot of multi-spiked QDs in ^{111}Cd in the HNO_3 2% matrix. The blue line indicates the best fit of the experimental data while the black dashed line is the ideal non-biased recovery plot ($C_{Cd@QD}^{predicted} = C_{Cd@QD}^{reference}$).

Selenium

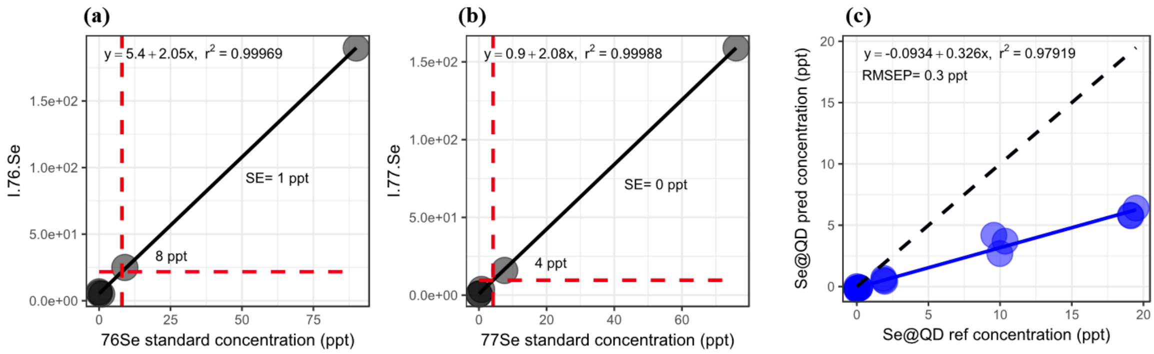

Figure S8. a) and b) Calibration plot of ^{76}Se and ^{77}Se. c) Recovery plot of multi-spiked QDs in ^{77}Se in the HNO_3 2% matrix. The blue line indicates the best fit of the experimental data while the black dashed line is the ideal non-biased recovery plot ($C_{Se@QD}^{predicted} = C_{Se@QD}^{reference}$).

The recovery plot of Se@QDs in the HNO_3 2% features a very low recovery rate (33%), which indicates a negative bias of 67%. The large bias was due to the low concentration of the Se@QDs, resulting in less precision during the HR-ICP-MS measurement. The same explanation is true for all recovery plots of Se coming from the QDs.

$NaNO_3$ 0.01M matrix

Zinc

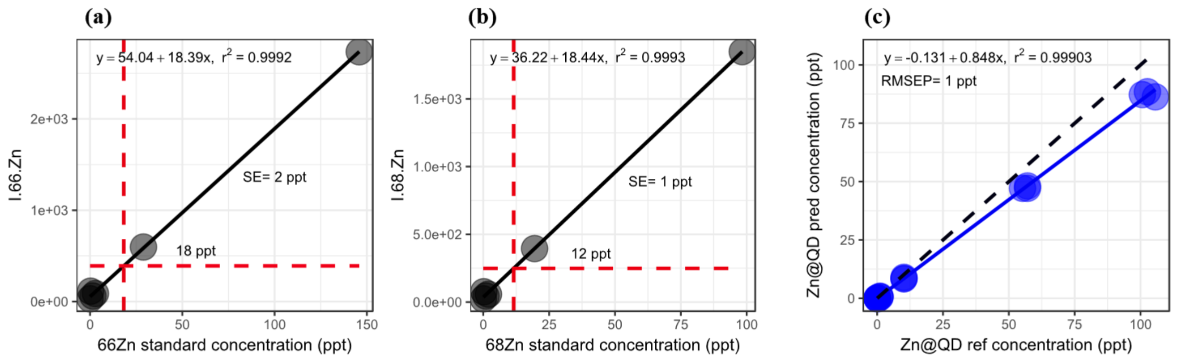

Figure S9. a) and b) Calibration plot of ^{66}Zn and ^{68}Zn. c) Recovery plot of multi-spiked QDs in ^{68}Zn in the $NaNO_3$ 0.01M matrix. The blue line indicates the best fit of the experimental data while the black dashed line is the ideal non-biased recovery plot ($C_{Zn@QD}^{predicted} = C_{Zn@QD}^{reference}$).

Cadmium

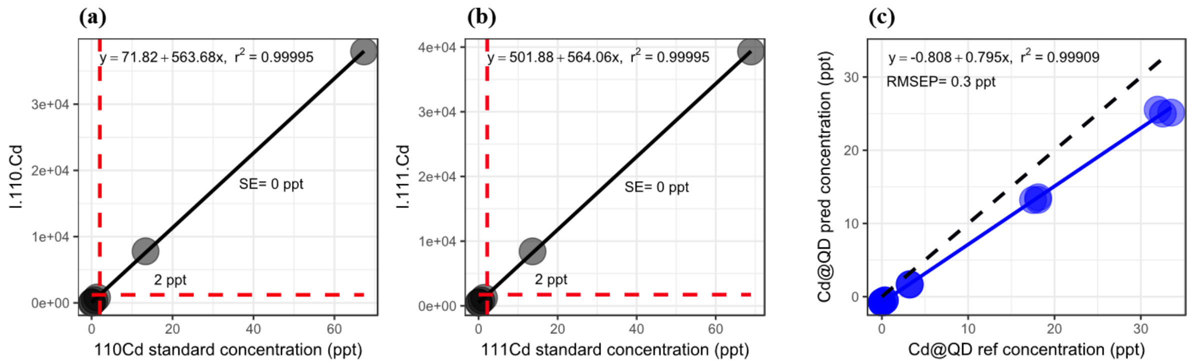

Figure S10. a) and b) Calibration plot of ^{110}Cd and ^{111}Cd. c) Recovery plot of multi-spiked QDs in ^{111}Cd in the $NaNO_3$ 0.01M matrix. The blue line indicates the best fit of the experimental data while the black dashed line is the ideal non-biased recovery plot ($C_{Cd@QD}^{predicted} = C_{Cd@QD}^{reference}$).

Saliva matrix

Zinc

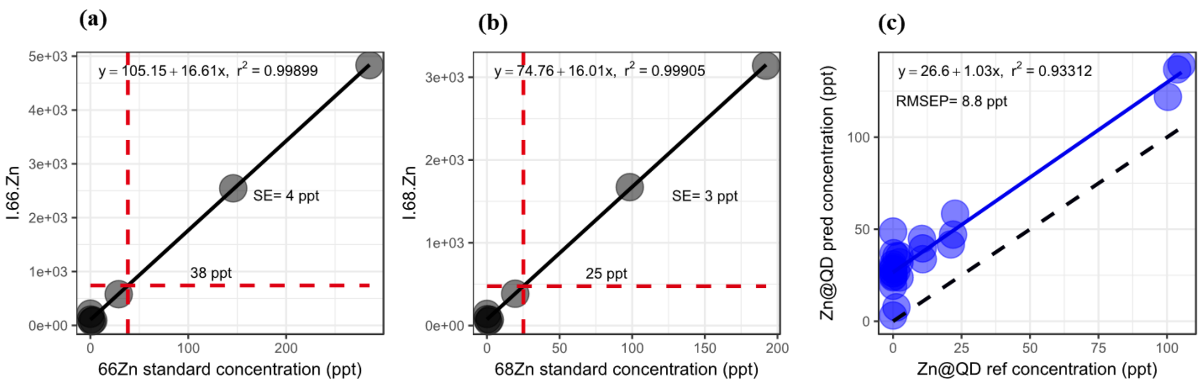

Figure S11. a) and b) Calibration plot of ^{66}Zn and ^{68}Zn. c) Recovery plot of multi-spiked QDs in ^{68}Zn in the saliva matrix (50-fold dilution). The blue line indicates the best fit of the experimental data while the black dashed line is the ideal non-biased recovery plot ($C^{predicted}_{Zn@QD} = C^{reference}_{Zn@QD}$).

Cadmium

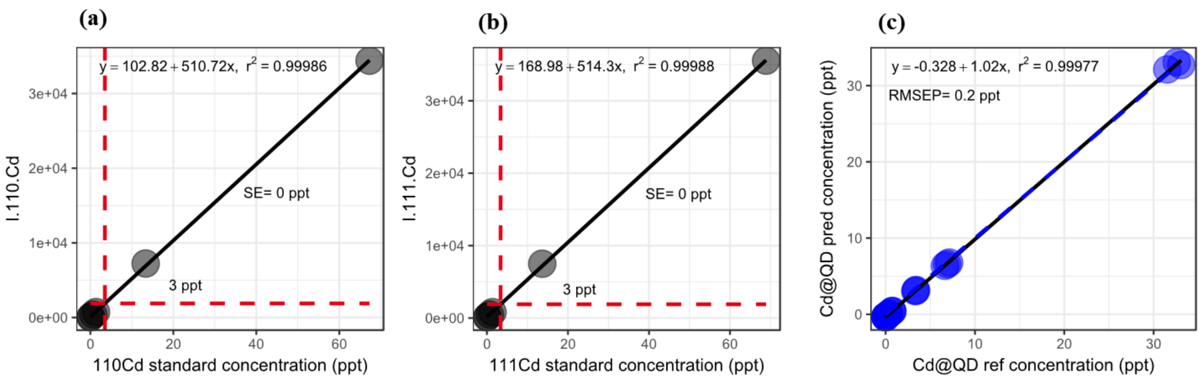

Figure S12. a) and b) Calibration plot of ^{110}Cd and ^{111}Cd. c) Recovery plot of multi-spiked QDs in ^{111}Cd in the saliva matrix (50-fold dilution). The blue line indicates the best fit of the experimental data while the black dashed line is the ideal non-biased recovery plot ($C^{predicted}_{Cd@QD} = C^{reference}_{Cd@QD}$).

Selenium

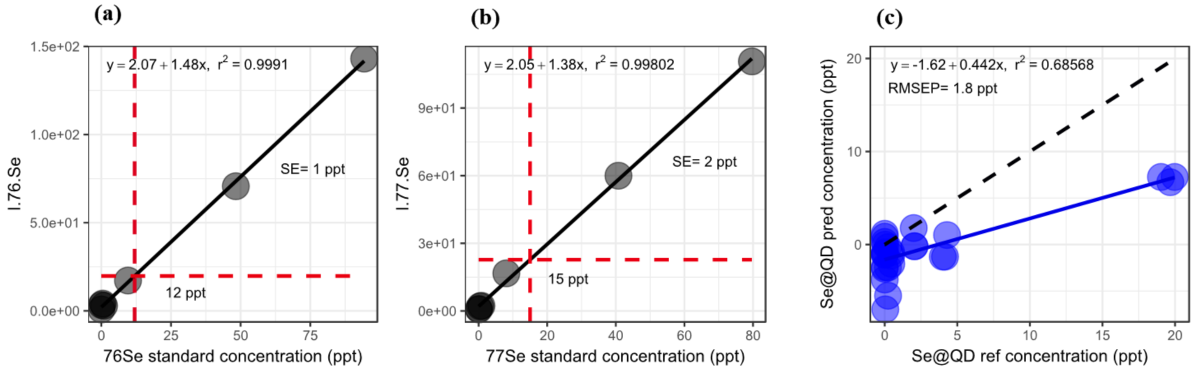

Figure S13. a) and b) Calibration plot of ^{76}Se and ^{77}Se. c) Recovery plot of multi-spiked QDs in ^{77}Se in the saliva matrix. The blue line indicates the best fit of the experimental data while the black dashed line is the ideal non-biased recovery plot ($C_{Se@QD}^{predicted} = C_{Se@QD}^{reference}$).

The recovery plot of Se@QDs in saliva matrix features a very low recovery rate (44%), which indicates a negative bias of 56%. The large bias was due to the low concentration of the Se@QDs, resulting in less precision during the HR-ICP-MS measurement. The same explanation is true for all recovery plots of Se coming from the QDs.

Urine matrix

Zinc

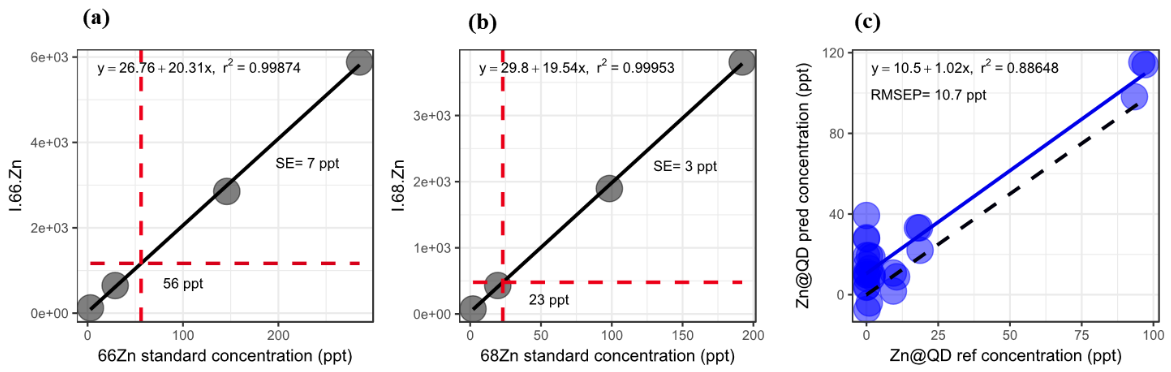

Figure S14. a) and b) Calibration plot of ^{66}Zn and ^{68}Zn. c) Recovery plot of multi-spiked QDs in ^{68}Zn in the urine matrix (50-fold dilution). The blue line indicates the best fit of the experimental data while the black dashed line is the ideal non-biased recovery plot ($C^{predicted}_{Zn@QD} = C^{reference}_{Zn@QD}$).

Cadmium

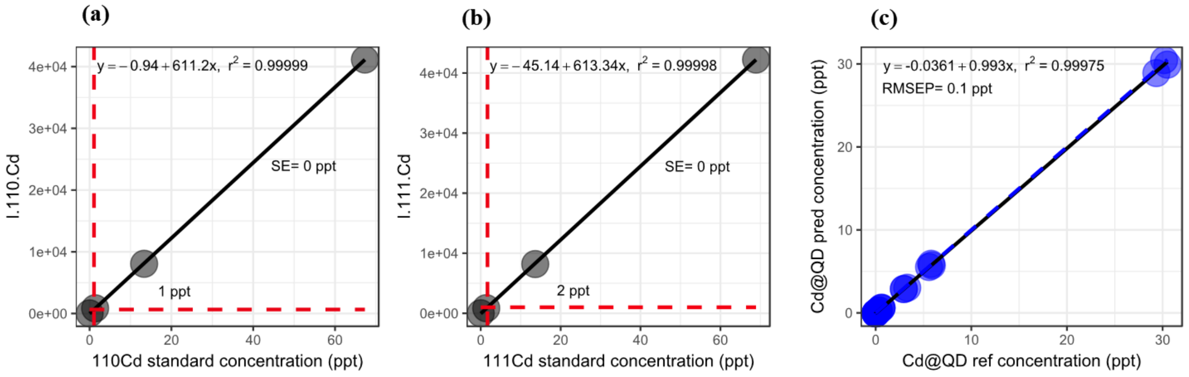

Figure S15. a) and b) Calibration plot of ^{110}Cd and ^{111}Cd. c) Recovery plot of multi-spiked QDs in ^{111}Cd in the urine (50-fold dilution). The blue line indicates the best fit of the experimental data while the black dashed line is the ideal non-biased recovery plot ($C^{predicted}_{Cd@QD} = C^{reference}_{Cd@QD}$).

Selenium

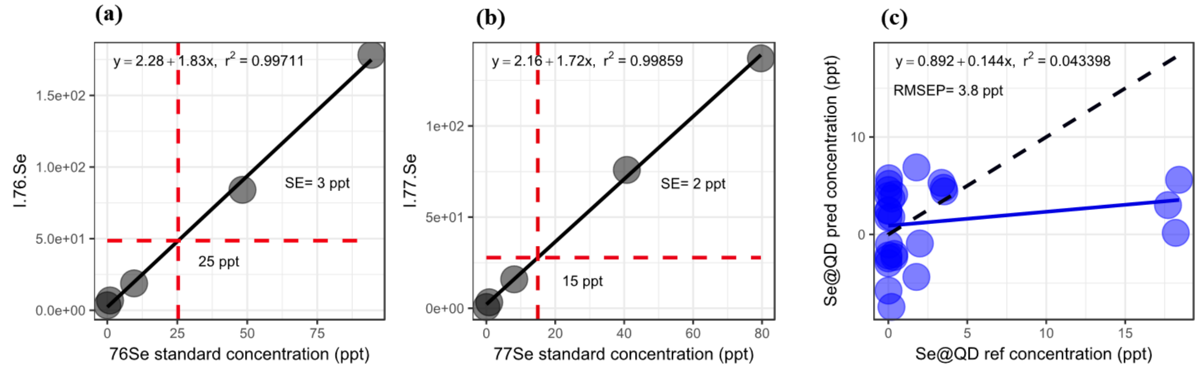

Figure S16. a) and b) Calibration plot of ^{76}Se and ^{77}Se. c) Recovery plot of multi-spiked QDs in ^{77}Se in the urine matrix (50-fold dilution). The blue line indicates the best fit of the experimental data while the black dashed line is the ideal non-biased recovery plot ($C_{Se@QD}^{predicted} = C_{Se@QD}^{reference}$).

The recovery plot of Se@QDs in urine matrix features a very low recovery rate (14%), which indicates a negative bias of 86%. The large bias was due to the low concentration of the Se@QDs, resulting in less precision during the HR-ICP-MS measurement. The same explanation is true for all recovery plots of Se coming from the QDs.

Blood plasma matrix

Zinc

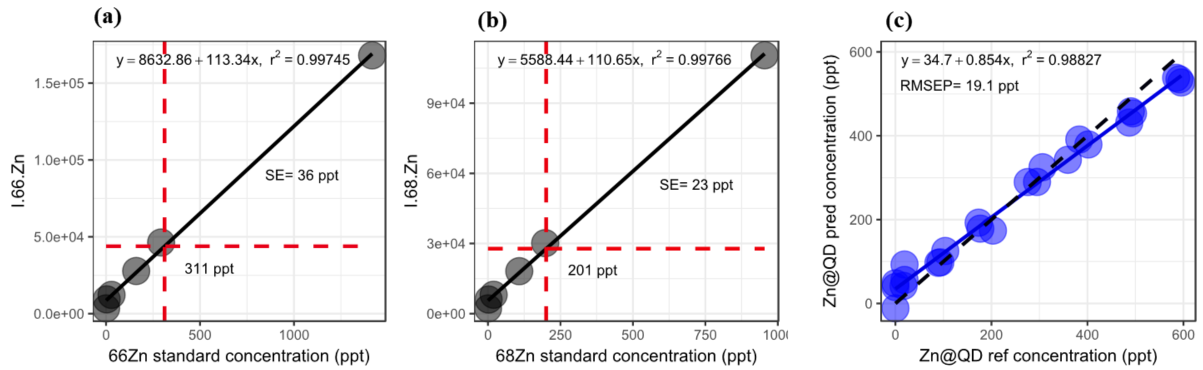

Figure S17. a) and b) Calibration plot of ^{66}Zn and ^{68}Zn. c) Recovery plot of multi-spiked QDs in ^{68}Zn in the plasma matrix (50-fold dilution). The blue line indicates the best fit of the experimental data while the black dashed line is the ideal non-biased recovery plot ($C^{predicted}_{Zn@QD} = C^{reference}_{Zn@QD}$).

Cadmium

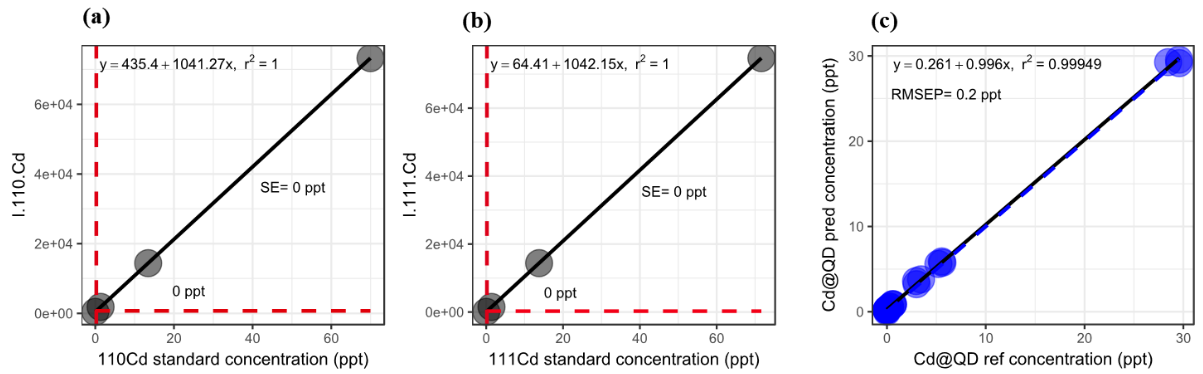

Figure S18. a) and b) Calibration plot of ^{110}Cd and ^{111}Cd. c) Recovery plot of multi-spiked QDs in ^{111}Cd in the plasma matrix (50-fold dilution). The blue line indicates the best fit of the experimental data while the black dashed line is the ideal non-biased recovery plot ($C^{predicted}_{Cd@QD} = C^{reference}_{Cd@QD}$).

Selenium

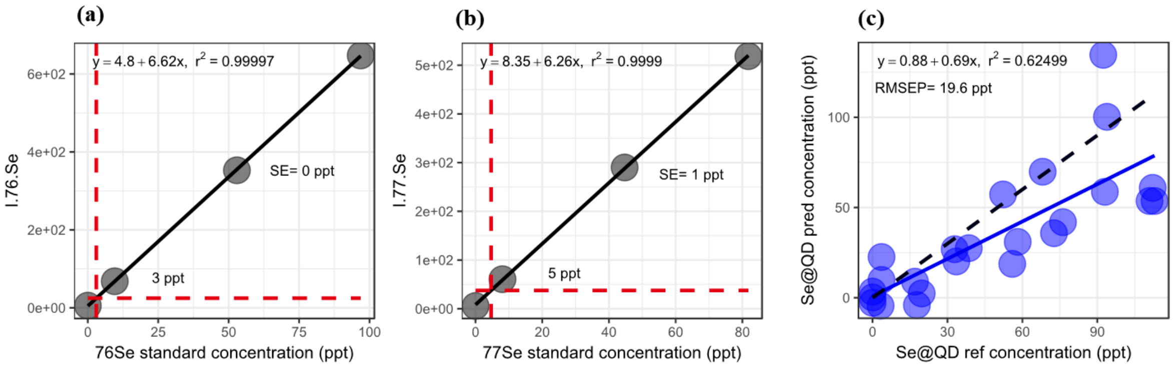

Figure S19. a) and b) Calibration plot of ^{76}Se and ^{77}Se. c) Recovery plot of multi-spiked QDs in ^{77}Se in the plasma (50-fold dilution). The blue line indicates the best fit of the experimental data while the black dashed line is the ideal non-biased recovery plot ($C_{Se@QD}^{predicted} = C_{Se@QD}^{reference}$).

DPBS matrix

Zinc

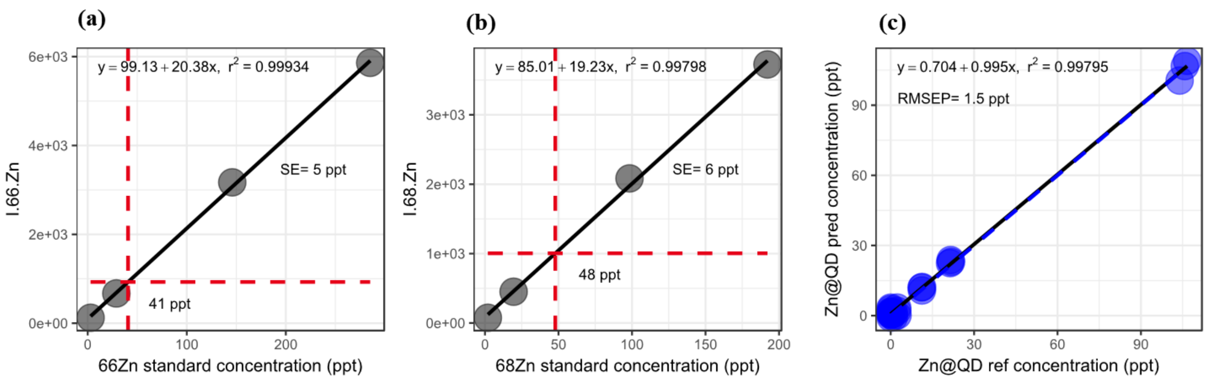

Figure S20. a) and b) Calibration plot of ^{68}Zn and ^{66}Zn. c) Recovery plot of multi-spiked QDs in ^{68}Zn in the DPBS matrix (50-fold dilution). The blue line indicates the best fit of the experimental data while the black dashed line is the ideal non-biased recovery plot ($C_{Zn@QD}^{predicted} = C_{Zn@QD}^{reference}$).

Cadmium

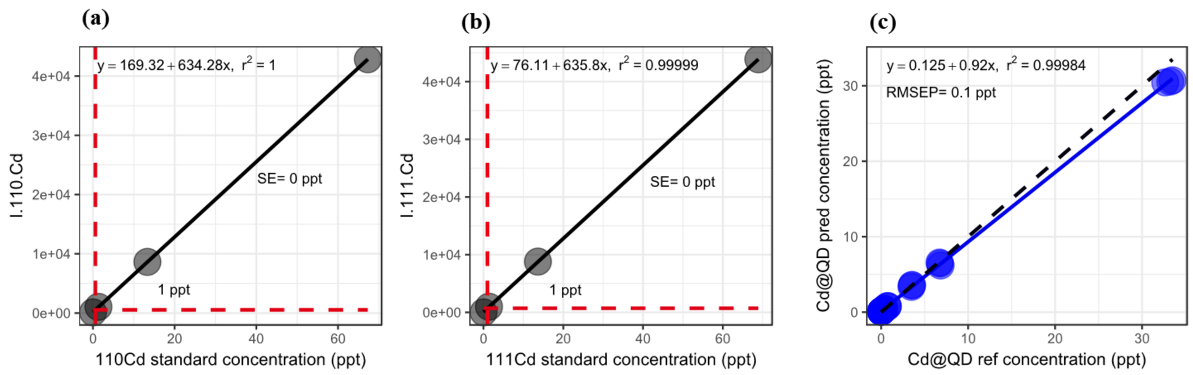

Figure S21. a) and b) Calibration plot of ^{110}Cd and ^{111}Cd. c) Recovery plot of multi-spiked QDs in ^{111}Cd in the DPBS matrix (50-fold dilution). The blue line indicates the best fit of the experimental data while the black dashed line is the ideal non-biased recovery plot ($C_{Cd@QD}^{predicted} = C_{Cd@QD}^{reference}$).

Selenium

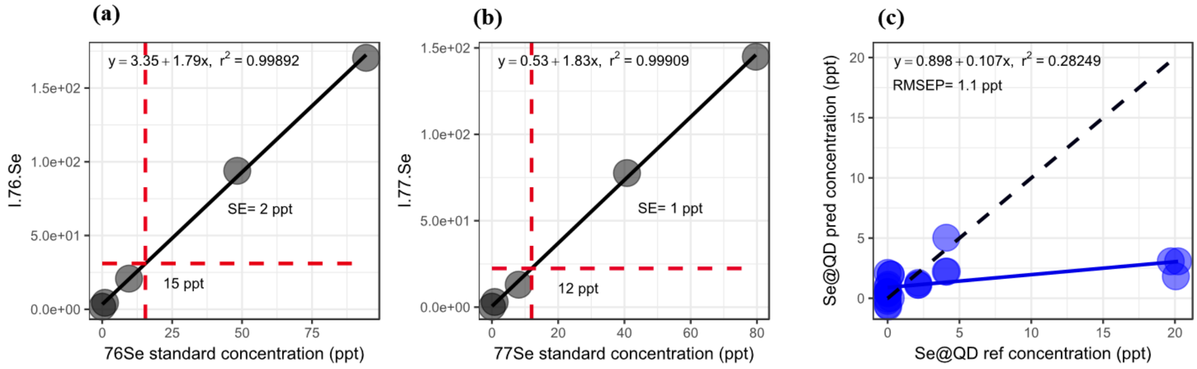

Figure S22. a) and b) Calibration plot of ^{76}Se and ^{77}Se. c) Recovery plot of multi-spiked QDs in ^{77}Se in the DPBS matrix (50-fold dilution). The blue line indicates the best fit of the experimental data while the black dashed line is the ideal non-biased recovery plot ($C_{Se@QD}^{predicted} = C_{Se@QD}^{reference}$).

The recovery plot of Se@QDs in DPBS matrix features a very low recovery rate (11%), which indicates a negative bias of 89%. The large bias was due to the low concentration of the Se@QDs, resulting in less precision during the HR-ICP-MS measurement.

Recovery plots: Bias

Table S4. Bias of the QDs concentrations recovery plots. n.a: not available.

Bias of the recovery plots			
**Matrix**	**Zn**	**Cd**	**Se**
HNO_3	-18%	-15%	-67%
$NaNO_3$	-15%	-21%	*n.a.*
Saliva	+3%	+2%	-56%
Urine	+2%	-0.7%	-86%
Plasma	-15%	-0.4%	-21%
DPBS	-0.5%	-8%	-89%

All the recovery plots of Se@QDs feature very low recovery rates indicating negative biases larger than 67%, except for the plasma matrix (which features a negative bias of 21%). The large bias was due to the low concentration of the Se@QDs, resulting in less precision during the HR-ICP-MS measurement. The better bias of Se@QDs recovery plots observed in the plasma matrix was thanks to the higher concentrations used in the additional experiment (where the concentration of Zn_{QDs} = 1 to 30 ppb, resulting in the concentration of Se_{QDs} = 190 to 5700 ppt).

t table

Table S5. *t* Table. Source: San Jose State University.

t Table

cum. prob	$t_{.50}$	$t_{.75}$	$t_{.80}$	$t_{.85}$	$t_{.90}$	$t_{.95}$	$t_{.975}$	$t_{.99}$	$t_{.995}$	$t_{.999}$	$t_{.9995}$
**one-tail**	**0.50**	**0.25**	**0.20**	**0.15**	**0.10**	**0.05**	**0.025**	**0.01**	**0.005**	**0.001**	**0.0005**
**two-tails**	**1.00**	**0.50**	**0.40**	**0.30**	**0.20**	**0.10**	**0.05**	**0.02**	**0.01**	**0.002**	**0.001**
**df**											
1	0.000	1.000	1.376	1.963	3.078	6.314	12.71	31.82	63.66	318.31	636.62
2	0.000	0.816	1.061	1.386	1.886	2.920	4.303	6.965	9.925	22.327	31.599
3	0.000	0.765	0.978	1.250	1.638	2.353	3.182	4.541	5.841	10.215	12.924
4	0.000	0.741	0.941	1.190	1.533	2.132	2.776	3.747	4.604	7.173	8.610
5	0.000	0.727	0.920	1.156	1.476	2.015	2.571	3.365	4.032	5.893	6.869
6	0.000	0.718	0.906	1.134	1.440	1.943	2.447	3.143	3.707	5.208	5.959
7	0.000	0.711	0.896	1.119	1.415	1.895	2.365	2.998	3.499	4.785	5.408
8	0.000	0.706	0.889	1.108	1.397	1.860	2.306	2.896	3.355	4.501	5.041
9	0.000	0.703	0.883	1.100	1.383	1.833	2.262	2.821	3.250	4.297	4.781
10	0.000	0.700	0.879	1.093	1.372	1.812	2.228	2.764	3.169	4.144	4.587
11	0.000	0.697	0.876	1.088	1.363	1.796	2.201	2.718	3.106	4.025	4.437
12	0.000	0.695	0.873	1.083	1.356	1.782	2.179	2.681	3.055	3.930	4.318
13	0.000	0.694	0.870	1.079	1.350	1.771	2.160	2.650	3.012	3.852	4.221
14	0.000	0.692	0.868	1.076	1.345	1.761	2.145	2.624	2.977	3.787	4.140
15	0.000	0.691	0.866	1.074	1.341	1.753	2.131	2.602	2.947	3.733	4.073
16	0.000	0.690	0.865	1.071	1.337	1.746	2.120	2.583	2.921	3.686	4.015
17	0.000	0.689	0.863	1.069	1.333	1.740	2.110	2.567	2.898	3.646	3.965
18	0.000	0.688	0.862	1.067	1.330	1.734	2.101	2.552	2.878	3.610	3.922
19	0.000	0.688	0.861	1.066	1.328	1.729	2.093	2.539	2.861	3.579	3.883
20	0.000	0.687	0.860	1.064	1.325	1.725	2.086	2.528	2.845	3.552	3.850
21	0.000	0.686	0.859	1.063	1.323	1.721	2.080	2.518	2.831	3.527	3.819
22	0.000	0.686	0.858	1.061	1.321	1.717	2.074	2.508	2.819	3.505	3.792
23	0.000	0.685	0.858	1.060	1.319	1.714	2.069	2.500	2.807	3.485	3.768
24	0.000	0.685	0.857	1.059	1.318	1.711	2.064	2.492	2.797	3.467	3.745
25	0.000	0.684	0.856	1.058	1.316	1.708	2.060	2.485	2.787	3.450	3.725
26	0.000	0.684	0.856	1.058	1.315	1.706	2.056	2.479	2.779	3.435	3.707
27	0.000	0.684	0.855	1.057	1.314	1.703	2.052	2.473	2.771	3.421	3.690
28	0.000	0.683	0.855	1.056	1.313	1.701	2.048	2.467	2.763	3.408	3.674
29	0.000	0.683	0.854	1.055	1.311	1.699	2.045	2.462	2.756	3.396	3.659
30	0.000	0.683	0.854	1.055	1.310	1.697	2.042	2.457	2.750	3.385	3.646
40	0.000	0.681	0.851	1.050	1.303	1.684	2.021	2.423	2.704	3.307	3.551
60	0.000	0.679	0.848	1.045	1.296	1.671	2.000	2.390	2.660	3.232	3.460
80	0.000	0.678	0.846	1.043	1.292	1.664	1.990	2.374	2.639	3.195	3.416
100	0.000	0.677	0.845	1.042	1.290	1.660	1.984	2.364	2.626	3.174	3.390
1000	0.000	0.675	0.842	1.037	1.282	1.646	1.962	2.330	2.581	3.098	3.300
z	0.000	0.674	0.842	1.036	1.282	1.645	1.960	2.326	2.576	3.090	3.291
	0%	50%	60%	70%	80%	90%	95%	98%	99%	99.8%	99.9%
	**Confidence Level**										

Statistic *t* values calculated in all matrices

The statistic t values calculated are valid for a two-tail test. The probabilities greater than 0.001 (P > 0.001, *i.e.* critical *t* < calculated statistic *t*) were considered as not significant (shaded in grey). For samples diluted prior to analysis, the estimated QD-LOQ from the *t*-test and the RMSEP were multiplied by the dilution factor.

Table S6. Statistic t values calculated in all matrices in the case of Zn. $Sample_1$ $sample_2$, $sample_3$, $sample_4$, and $sample_5$ correspond to C_{Zn}^{QD} ≈ 0.1, 1.0, 10, 50, and 100 ppt, respectively. As for plasma, C_{Zn}^{QD} ≈ 1000, 5000, 10000, 15000, and 20000 ppt, respectively. Higher concentrations than these mentioned values are not included in the table.

Zn	Critical *t*	Calculated statistic *t*								
Medium		Matrix blank *vs.* $Sample_1$	Matrix blank *vs.* $Sample_2$	Matrix blank *vs.* $Sample_3$	Matrix blank *vs.* $Sample_4$	Matrix blank *vs.* $Sample_5$	Dilution factor	Estimated $QD\text{-}LOQ_{Zn}$ (ppt)	Statistic *t* at $QD\text{-}LOQ_{Zn}$	RMSEP (ppt)
HNO_3 2%	12.924	1.14	8.28	14.06	24.47	106.27	1	**10**	13.91	**1**
$NaNO_3$ 0.01 M	8.61	-2.88	3.66	40.3	136.2	135.6	1	**7**	9.63	**1**
Saliva	8.61	0.79	-2.16	-0.01	-0.7	0.35	51.7	**2275**	8.613	**455**
Urine	8.61	-0.58	-0.01	-1.63	-0.9	-0.75	51.5	**5459**	8.614	**551**
Plasma	8.61	1.6	3.93	8.05	12.57	14.65	52.9	**10598**	8.617	**973**
DPBS	8.61	2.56	0.5	0.73	0.11	1.24	49.6	**446**	8.666	**74**

Table S7. Statistic t values calculated in all matrices in the case of Cd. $Sample_1$ $sample_2$, $sample_3$, $sample_4$, and $sample_5$ to $C_{Cd}^{QD} \approx 0.03$, 0.3, 3.0, 15.7, and 31.6 ppt, respectively.

Cd	Critical *t*	Calculated statistic *t*								
Medium		Matrix blank *vs.* $Sample_1$	Matrix blank *vs.* $Sample_2$	Matrix blank *vs.* $Sample_3$	Matrix blank *vs.* $Sample_4$	Matrix blank *vs.* $Sample_5$	Dilution factor	Estimated QD-LOQ_{Cd} (ppt)	Statistic *t* at QD-LOQ_{Cd}	RMSEP (ppt)
HNO_3 2%	8.61	3.61	21.01	170.14	38.62	94.34	1	**0.3**	21.01	**0.1**
$NaNO_3$ 0.01 M	8.61	5.73	55.88	107.23	119.02	160.10	1	**1.9**	8.66	**0.3**
Saliva	8.61	1.04	1.96	5.19	17.95	35.12	51.7	**20**	17.95	**7.8**
Urine	8.61	1.08	1.28	4.87	29.64	31.57	51.5	**16**	29.64	**7.2**
Plasma	8.61	1.88	2.21	3.39	8.06	12.8	52.9	**32**	12.8	**11**
DPBS	8.61	-0.04	0.03	3.5	20.32	36.69	49.6	**17**	20.32	**6**

Table S8. Statistic t values calculated in all matrices in the case of Se. $Sample_1$ $sample_2$, $sample_3$, $sample_4$, and $sample_5$ to $C_{Se}^{QD} \approx 0.02$, 0.2, 1.9, 9.5, and 19 ppt, respectively. As for plasma, $C_{Se}^{QD} \approx 190$, 950, 1900, 2850, and 3800 ppt, respectively. Higher concentrations than these mentioned values are not included in the table.

Se	Critical *t*	Calculated statistic *t*								
Medium		Matrix blank *vs.* $Sample_1$	Matrix blank *vs.* $Sample_2$	Matrix blank *vs.* $Sample_3$	Matrix blank *vs.* $Sample_4$	Matrix blank *vs.* $Sample_5$	Dilution factor	Estimated QD-LOQ_{Se} (ppt)	Statistic *t* at QD-LOQ_{Se}	RMSEP (ppt)
HNO_3 2%	8.61	-0.67	0.46	6.81	8.22	28.8	1	**6**	8.750	**0.4**
Saliva	8.61	0.75	-0.04	-0.45	-0.66	0.09	51.7	**1618**	8.626	**93**
Urine	8.61	0.36	-0.44	1.06	-0.27	-0.18	51.5	**8317**	8.613	**196**
Plasma	8.61	1.13	0.52	8.28	3.08	4.6	52.9	**7219**	8.616	**1009**
DPBS	8.61	-1.65	-1.1	-0.96	0.35	0.26	49.6	**3388**	8.613	**55**

ANOVA Test Tables

Impacts of background concentrations (BCs) of each tracer element, matrix dilution and ICP-MS resolution on the QD-LOQs were examined using the ANOVA methodology on the experimental data organized as presented in Table S9 and the models listed in Table S10.

Table S9. Experimental data submitted to ANOVA.

Matrix	Dilution factor	Element	BC (ppt)	ICP Resolution	Recovery	QD-LOQ (ppt)
HNO_3	1	Zn	0	4000	82	10
$NaNO_3$	1	Zn	0	4000	85	7
Saliva	51.7	Zn	222000	4000	103	2275
Urine	51.5	Zn	650000	4000	102	5459
Plasma	52.9	Zn	1020000	4000	85	10598
DPBS	49.6	Zn	7700	4000	99.5	446
HNO_3	1	Cd	0	300	85	0.31
$NaNO_3$	1	Cd	0	300	79	1.9
Saliva	51.7	Cd	500	300	102	20
Urine	51.5	Cd	300	300	99.3	16
Plasma	52.9	Cd	45	300	99.6	32
DPBS	49.6	Cd	0	300	92	17
HNO_3	1	Se	0	10000	33	6
Saliva	51.7	Se	3000	10000	44	1618
Urine	51.5	Se	40000	10000	14	8317
Plasma	52.9	Se	620000	10000	79	7291
DPBS	49.6	Se	1580	10000	11	3388

Table S10. Overview of ANOVA models examined to account for variabilities in recovery rates and QD-LOQs.

Model reference	Analytical expression	Dataset
M1	*Recovery rate ~ BC+ICP resolution*	Cd, Zn, Se
M2	*Recovery rate ~ ICP resolution*	Cd, Zn, Se
M3	*QD-LOQ ~ BC+dilution factor*	Zn, Cd
M4	*QD-LOQ ~ BC+dilution factor*	Zn
M5	*QD-LOQ ~ dilution factor+BC*	Cd

Table S11. Summary of ANOVA on model M1.

```
                Df  Sum Sq  Mean Sq  F value  Pr(>F)
BC               1     640      640    2.159  0.164
ICP.resolution   1   10438    10438   35.218  3.64e-05 ***
Residuals       14    4150      296
---
Signif. codes:  0 '***' 0.001 '**' 0.01 '*' 0.05 '.' 0.1 ' ' 1
```

Table S12. Summary of ANOVA on model M2.

```
                Df  Sum Sq  Mean Sq  F value  Pr(>F)
ICP.resolution   1    9514     9514    24.98  0.000159 ***
Residuals       15    5714      381
---
Signif. codes:  0 '***' 0.001 '**' 0.01 '*' 0.05 '.' 0.1 ' ' 1
```

Table S13. Summary of ANOVA on model M3.

```
                Df     Sum Sq    Mean Sq  F value   Pr(>F)
BC               1  116447648  116447648  785.703   4.55e-10 ***
dilution.factor  1       1937       1937    0.013   0.911
Residuals        9    1333874     148208
---
Signif. codes:  0 '***' 0.001 '**' 0.01 '*' 0.05 '.' 0.1 ' ' 1
```

Table S14. Summary of ANOVA on model M4.

```
                Df   Sum Sq  Mean Sq F value   Pr(>F)
BC               1 87283360 87283360 196.730 0.000785 ***
dilution.factor  1     3262     3262   0.007 0.937072
Residuals        3  1331016   443672
---
Signif. codes:  0 '***' 0.001 '**' 0.01 '*' 0.05 '.' 0.1 ' ' 1
```

Table S15. Summary of Linear Model between log QD-LOQ$_{Zn}$ and log BC.

```
Coefficients:
            Estimate   Std. Error   t value   Pr(>|t|)
(Intercept)  0.26179      0.21170     1.237      0.284
log.BC       0.63166      0.02083    30.330   7.04e-06 ***
---
Signif. codes:  0 '***' 0.001 '**' 0.01 '*' 0.05 '.' 0.1 ' ' 1

Residual standard error: 0.234 on 4 degrees of freedom
Multiple R-squared:  0.9957,    Adjusted R-squared:  0.9946
F-statistic: 919.9 on 1 and 4 DF,  p-value: 7.04e-06
```

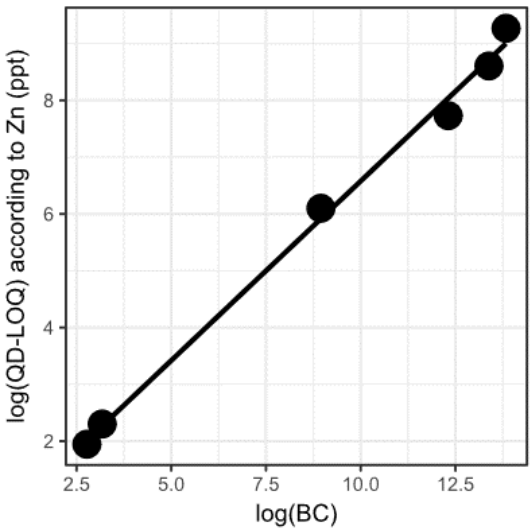

Figure S23. log QD-LOQ$_{Zn}$ *vs.* log BC. The summary is detailed in Table S15.

Table S16. Summary of ANOVA on model M5.

```
                 Df  Sum Sq Mean Sq F value  Pr(>F)
dilution.factor   1   558.6   558.6  13.481   0.035 *
BC                1    22.2    22.2   0.536   0.517
Residuals         3   124.3    41.4
---
Signif. codes:  0 '***' 0.001 '**' 0.01 '*' 0.05 '.' 0.1 ' ' 1
```

Table S17. Summary of Linear Model between QD-LOQ$_{Cd}$ and dilution factor.

```
Coefficients:
                 Estimate Std. Error t value Pr(>|t|)
(Intercept)       0.42175    4.64093   0.091   0.9333
dilution.factor   0.45536    0.12973   3.510   0.0392 *
---
Signif. codes:  0 '***' 0.001 '**' 0.01 '*' 0.05 '.' 0.1 ' ' 1

Residual standard error: 6.437 on 3 degrees of freedom
Multiple R-squared:  0.8237,    Adjusted R-squared:  0.7062
F-statistic: 7.008 on 2 and 3 DF,  p-value: 0.07402
```

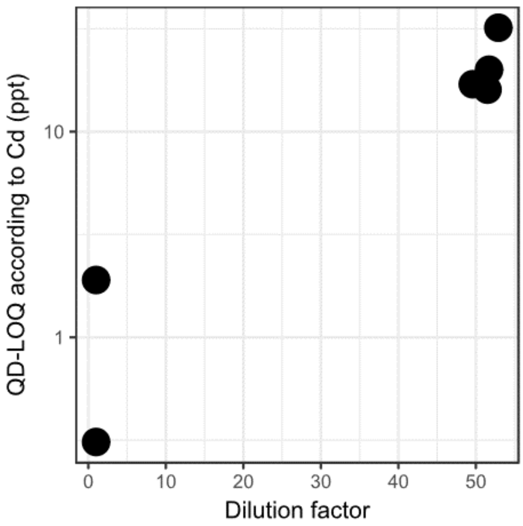

Figure S24. QD-LOQ$_{Cd}$ *vs.* dilution factor. The summary is detailed in Table S17.

QD-RLOQs comparison overview

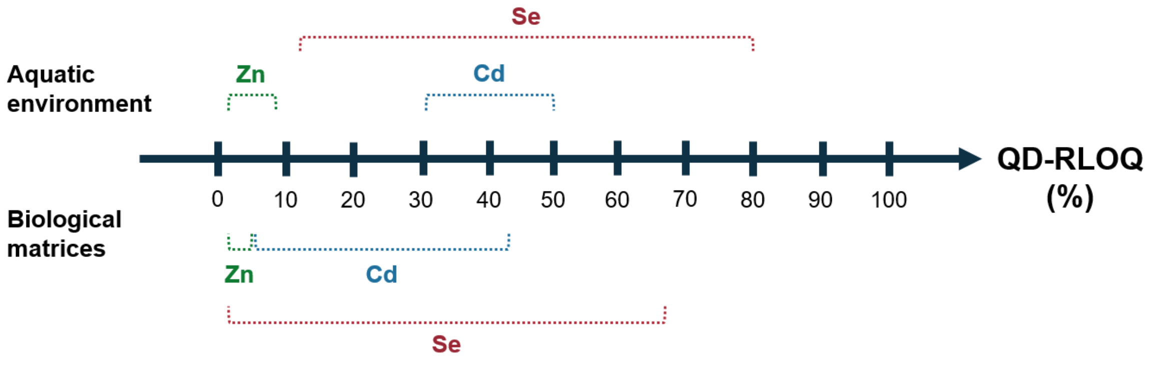

Figure S25. Comparison overview of the QD-RLOQs in aquatic and biological matrices.
